# Identification of chromosomal alpha-proteobacterial small RNAs by comparative genome analysis and detection in *Sinorhizobium meliloti *strain 1021

**DOI:** 10.1186/1471-2164-8-467

**Published:** 2007-12-19

**Authors:** Vincent M Ulvé, Emeric W Sevin, Angélique Chéron, Frédérique Barloy-Hubler

**Affiliations:** 1 CNRS UMR6061 Génétique et Développement, Groupe Modèles Génétiques, Université de Rennes 1, IFR140 GFAS, Faculté de médecine, 2 avenue du Professeur Léon Bernard, CS 34317, 35043 Rennes Cedex, France; 2 CNRS UMR6026 Interactions Cellulaires et Moléculaires, Groupe DUALS, Université de Rennes 1, IFR140 GFAS, Campus de Beaulieu, avenue du Général Leclerc, 35042 Rennes, France

## Abstract

**Background:**

Small untranslated RNAs (sRNAs) seem to be far more abundant than previously believed. The number of sRNAs confirmed in *E. coli *through various approaches is above 70, with several hundred more sRNA candidate genes under biological validation. Although the total number of sRNAs in any one species is still unclear, their importance in cellular processes has been established. However, unlike protein genes, no simple feature enables the prediction of the location of the corresponding sequences in genomes. Several approaches, of variable usefulness, to identify genomic sequences encoding sRNA have been described in recent years.

**Results:**

We used a combination of *in silico *comparative genomics and microarray-based transcriptional profiling. This approach to screening identified ~60 intergenic regions conserved between *Sinorhizobium meliloti *and related members of the alpha-proteobacteria sub-group 2. Of these, 14 appear to correspond to novel non-coding sRNAs and three are putative peptide-coding or 5' UTR RNAs (ORF smaller than 100 aa). The expression of each of these new small RNA genes was confirmed by Northern blot hybridization.

**Conclusion:**

Small non coding RNA (*sra*) genes can be found in the intergenic regions of alpha-proteobacteria genomes. Some of these *sra *genes are only present in *S. meliloti*, sometimes in genomic islands; homologues of others are present in related genomes including those of the pathogens *Brucella *and *Agrobacterium*.

## Background

Numerous DNA sequences giving rise to small non-coding RNAs (ncRNAs or sRNAs, ranging from size 50 to 250 nt for the vast majority of them) have been found in bacterial plasmids, phages, transposons and chromosomes. Estimates of the number of ncRNA genes in *E. coli *range from 50 to several hundred [1,2]. The first ncRNAs were detected in the 1960s by chance, discovered by direct labelling as being associated with proteins on migration gels or identified after random mutations. The abundance of bacterial genome sequence data has allowed gene-finding computer programs to annotate a large number of prokaryote sequences. However, although *de novo *annotation programs successfully identify and map protein-coding genes, they are not designed to identify ncRNA genes. Recently, the intergenic regions (IGRs) of selected bacteria and yeast genomes were systematically searched for ncRNA genes. These computational screenings involved a combination of criteria, including large gaps between protein-coding genes [3,4] even though in *Sulfolobus solfataricus*, 13 small RNAs (sRNAs) have been found encoded either within, or overlapping, annotated open reading frames [5]. Other criteria used are extended conservation between species [2,4,6], orphan promoter or terminator sequences [2,4,7], base-composition signatures [8,9], and conserved secondary structures in deduced RNA sequences [10-13] even if not always significant [14]. Recently, ncRNA research algorithms have been developed including some or all of these criteria [15-18]. Supplementary *in vivo *experiments involving for example studies of expression patterns by Northern blotting or microarray testing are still essential to confirm that the sRNA candidate genes are indeed transcribed; such studies also provide information about temporal expression patterns, potential precursor forms and degradation products. In addition to *in silico *analysis, experimental *in vivo *RNomics were also developped (for example RACE and SELEX) [19-21].

ncRNAs are involved in a great variety of processes including chromosome replication and cell division (*dicF *[22]), transcriptional regulation (6S RNA [23]), RNA processing (RNase P or *rnpB*, [24]), mRNA stability and translation (antisense sequences such as *spot42 *[25]), protein stability (*tmRNA*, [26]) and transport (*4.5S *or *ffs*, [27]), stress adaptation (for example *oxyS *[28]), transition from growth to stationary phase (*dsrA*, *rprA *[29,30]), quorum sensing and virulence (*qrr *[31]), plasmid copy number control (RNAI and RNAIII [32,33]), carbon storage (*csrBC *[34]), and oligopeptide transport (*gcvB *[35]). Some provide housekeeping functions and others are regulators of stress gene expression, e.g. sRNAs modulating the bacterial cell surface by antisensing outer membrane protein (*omp*) genes (such as *micF *and *micC*, reviewed in [36,37]). In many cases, ncRNAs are associated with proteins that enhance their function (Hfq [38], SmpB [39]). Their mechanisms of action can be grouped into three main categories: antisense by base-pairing with another RNA/DNA molecule (*oxyS*), RNA structure mimicry (6S, *tmRNA*) and catalytic functions (*rnpB*). These categories are not exclusive (some ncRNAs can be classified in more than one category). Also, not all mechanisms of action are known and it is likely that some ncRNAs act in ways that have not yet been described.

*Sinorhizobium meliloti *(formerly *Rhizobium meliloti) *is a common Gram-negative soil bacterium that lives symbiotically on the roots of certain genera of leguminous plants (including *Medicago *and *Melilotus*). The bacterium enters the root tissue through infection threads and forms nodules, inside which it converts atmospheric nitrogen into ammonia. In return, the plant provides an energy source for the bacteria. Excess nitrogen remains in the soil, potentially reducing the need for fertilisers. *S. meliloti *is one of the best known *Rhizobia*; it has been extensively studied by numerous groups worldwide and is readily amenable to genetic studies. Like many other members of the alpha-proteobacteria, this fast growing *Rhizobium *possesses a multipartite genome: a 3.65-Mb chromosome and two megaplasmids, pSymA (1.35 Mb) and pSymB (1.68 Mb) [40]. Its genome shares various fundamental similarities with those of some other symbiotic bacteria and various plant (*Agrobacterium*) and animal pathogens (*Bartonella*, *Brucella*). As for most sequenced prokaryotic genomes, the annotation of the *S. meliloti *genome has led to the prediction of protein-coding genes but yielded very little information about non-coding RNA genes. When the genome sequence of the *S. meliloti *strain 1021 was completed [40], its RNome was only composed of three identical rRNA operons, 54 tRNAs (53 decoding the standard 20 aa and one selenocysteine tRNA), and a single annotated ncRNA, *ssrA *(tmRNA or *smc04478*). The characterisation and expression patterns of this RNA gene were recently published [41]. Two other ncRNAs are described in dedicated databases [27,42] one matching *rnpB*, the RNA component of the ubiquitous RNAse P ribonucleoprotein enzyme, and the other *ffs *(4.5S RNA), the RNA constituent of the signal recognition particle (SRP). Recent work by MacLellan with *S. meliloti *strain 1021 [43] and Izquierdo with strain GR4 [44] also describes *ctRNAs *(counter-transcribed-RNA) involved in plasmid incompatibility. In addition, 5' untranslated regions (UTR) of mRNAs that act as "riboswitches" have also been predicted in *S. meliloti*. Those cited by the Rfam database [45,46] sense concentrations of vitamins B2 (RFN, upstream from *ribH2*) and B12 (cobalamin, upstream from *cobP*, *smb20056*, *smc00982*, *smc00166 *and between *smb20555*-*smb20556*), of thiamine (THI, in 5' of *thiD, thiC *and *smc03869*) and of glycine (5' end of *gcvT*). Corbino *et al*. [47] also describe riboswitches for methionine (SAM, in front of *metA *and *metZ*) and serine (*serC*), as well as elements upstream of *suhB*, *smc02983 *(*speF*) and *smc03839 *(*ybhL*). No lysine, leucine, threonine or tryptophan riboswitches have been predicted in *S. meliloti *or in related alpha-proteobacteria. Finally, a repression of heat shock gene expression (ROSE) element is also annotated in the RFAM database, in the 5' translated region of *smb21295 *(ibpA-like).

The purpose of this study was to discover unidentified small RNA genes in the chromosome of *S. meliloti *and related alpha-proteobacteria. We used computational comparative genomic prediction of sRNA gene candidates combined with expression profiling using dedicated microarrays, followed by Northern hybridization. This approach revealed 17 previously unidentified sRNAs, eight of which are widely conserved in the alpha-proteobacteria phylum. These analyses suggest that, like other free-living bacteria, alpha-proteobacteria encode numerous sRNAs, although their number and their nature may differ between species.

## Results and discussion

### Profile of small RNAs in *S. meliloti*

**Figure 1:**
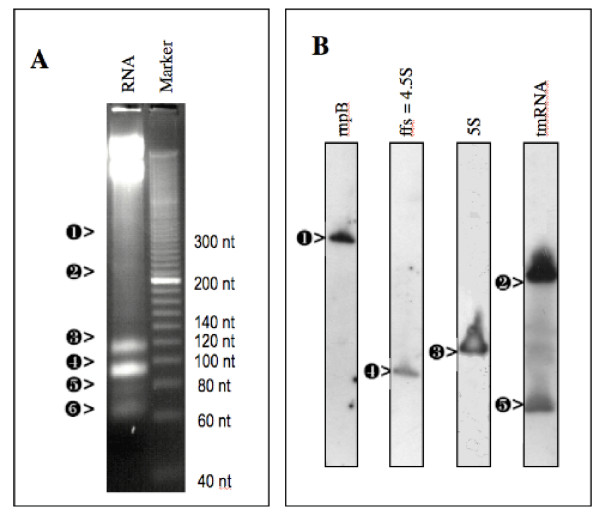
**Identification of small "known" RNAs in the *S. meliloti *chromosome**. A: Migration of 10 μg of *S. meliloti *total RNA in a 2.5% high-resolution agarose gel (Sigma) stained with ethidium bromide. B: 5 μg of total RNA from *S. meliloti *strain 1021 was analyzed by Northern blotting hybridization with specific oligonucleotides [see Additional file 8]; the molecular sizes were calculated in nucleotides (nt):  > rnpB (372 nt),  > tmRNA 3' end (204 nt),  > 5S (120 nt),  > 4.5S (95 nt), and  > tmRNA 5' end (82 nt) [39]. Band  > was not hybridized but its size is consistent with it being a tRNA. Exposure times were optimized for each panel and signal intensity does not indicate relative abundance of ncRNAs.

Staining of total RNA from bacteria resolved on gels reveals abundant sRNAs [48-52]. We used this method to analyse *S. meliloti *small RNA (<400 nt: Figure 1A). Four intense bands were detected, three of which correspond to the sizes predicted for 5S RNA (band 5), 4.5S RNA (band 2), and the 5' end of tmRNA (band 4, [41]). Band 6 (≈70 nt) corresponds to a length and migration profile compatible with tRNAs. No RNAs migrating faster than 70 nt were detected. Two less intense bands (1 and 3) were observed and were compatible with sizes predicted for *rnpB *RNA and for the 3' end of tmRNA [41]. Northern blotting confirmed the identity of each of these bands (Figure 1B). Surprisingly, no additional RNAs were visualized with either RNA extraction method (Trizol™ or Qiagen™, not shown) or stress (see conditions in Material and Methods section, data not shown). This experiment indicated that small RNAS do not constitute an abundant class of RNAs in *S. meliloti*, in contrast to what has been observed in marine Cyanobacteria and in *Staphylococcus aureus *[50-52]. As a consequence, we could not employ direct elution and sequencing to identify new sRNAs [53] in *S. meliloti*. We thus used computational prediction to identify candidates for further testing.

### Known sRNAs of *S. meliloti*: distinguishable biases?

**Figure 2:**
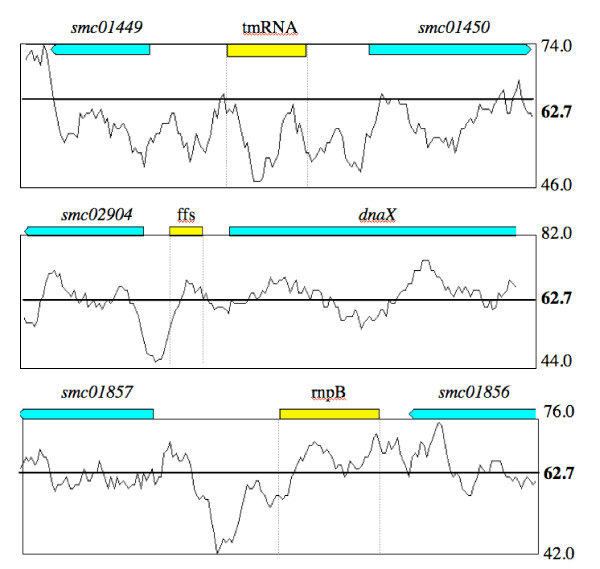
**Nucleotide bias analyses of known *S. meliloti *RNAs**. Local variations of GC content (%) with a window size of 100 bp, in *S. meliloti *genomic regions around *tmRNA*, *ffs *and *rnpB*. The y-axis represents the minimal, maximal and mean values of the percentage of GC.

ncRNAs in AT-rich hyperthermophiles can be located on the basis of local variations in genomic base composition [10,11]. Although the *S. meliloti *chromosome is GC-rich (62.7%), we scanned the regions containing *tmRNA*, *rnpB *and *ffs *(Figure 2) for base composition signals. Unlike AT-rich genomes, no difference was observed between sRNA and background genomic sequences (Table 1). BLAST alignments of the three sRNAs against all sequenced bacteria revealed significant similarities with sequences from the alpha-2 subgroup of the class Proteobacteria. However maximum likelihood trees (see Additional file 1) showed that most identical sequences were from different genomes (*R. etli *and *leguminosarum *for *ffs *and *tmRNA; Mezorhizobium *for *rnpB*). To conclude, no apparent dominant bias was observed for the three known *S. meliloti *ncRNAs, except primary sequence conservation in IGRs of related alpha-proteobacteria genomes (especially subgroup-2).

**Table 1:** Selected features of "known" ncRNAs of *S. meliloti*

Name	Strand	% IGR	A%	C%	G%	T%	GC%	GC-AT skew
4.5S = ffs	+/Leading	34.5	18.2	33.7	33.7	14.4	67.4	0.00–11.76
tmRNA	+/Lagging	45.1	24.3	28.0	28.4	19.3	56.4	0.55–11.43
rnpB	-/Leading	44.6	22.1	30.5	36.6	10.8	67.1	9.09–33.85

### Selection of sRNA candidate genes in the *S. meliloti *chromosome

Most bacterial sRNA searches were conducted using the *E. coli *genome as a reference, so we first compiled the sRNAs available in the Rfam database for that organism (strain K12, riboswitch/cis-regulatory genes excluded) to try to identify sRNA orthologs using BLAST alignments (e-value < 1) against the complete *S. meliloti *chromosome sequence. No significant similarities were detected except for *4.5S *and *rnpB *(see Additional file 2). *ssrA *(tmRNA) was not detected, presumably because the *S. meliloti *tmRNA is in a two-piece "permuted" form [41]. Surprisingly, the "ubiquitous" *ssrS *(6S RNA) was not detected either. Similar analysis with *Bacillus *and *Pseudomonas *sRNAs [RFAM] gave no further hits. This first approach confirmed a key point: most sRNAs cannot be detected by inter-phylum primary sequence identity.

RNA-specific tools described in the literature were then assessed. However, most rely on preliminary structural knowledge, knowledge which is poor for this phylum. Thus only a subset of these tools was tested: those reported to be able to detect new sRNA gene candidates in prokaryotic species. ISI (Intergenic Sequence Inspector, [54]) relies on inappropriate filters (in particular GC%), and was far too sensitive if the filters were disabled (close to 300 candidates). The use of QRNA [7] requires preliminary alignments. We tested it by aligning the 61 sRNAs annotated in the *E. coli *(K12) genome against *S. meliloti's *chromosome, but only *4.5S *and *rnpB *were predicted by this tool. Finally, we assessed sRNApredict2 [18], which combines commonly sRNA-associated genetic features; it was used to search the *S. meliloti *chromosome IGRs for sRNAs, by comparison with *A. tumefaciens *and *R. etli*. As shown in the output tables (see Additional file 3), 13 sRNAs were predicted, seven being common to the two genomes compared. However, a detailed analysis of each candidate revealed that only one (*pred1*) was likely to correspond to a genuine small RNA gene whereas the others are almost certainly 5' UTRs (in view of the small distances and nature of the surrounding genes) or repeated elements (RIME, Sm- [55,56]).

We ended up selecting for further analysis: the IGRs containing the origin of replication *oriC *(as a negative control) and the *tmRNA*, *rnpB *and *4.5S *RNA genes; 22 IGRs longer than 450 nt; 13 IGRs showing short sequence identity in alpha-proteobacteria (see Additional file 10); and 28 IGRs selected using the Artemis Comparison Tool (ACT) with related genomes (see Additional file 11). Indeed, the sequence identities marked by the ACT were mostly confined to actual open reading frames (ORFs), even between two close chromosomes (*S. meliloti *and *A. tumefaciens*). Although hits in intergenic regions were extremely rare (less than 40), all three *S. meliloti *known sRNAs (*tmRNA*, *4.5S *and *rnpB*) were included in these hits: we thus concluded similar ACT-visualized IGRs represented good sRNA gene candidates. Among these IGRs, those containing large repeats (Sm-1 to Sm-5) or RIMEs were however excluded, as were IGRs smaller than 50 nt (to avoid inclusion of 5' or 3' UTR elements).

This selection accounted for 67 candidate IGRs (see Additional file 4), which throughout this paper shall be referred to as *sra*, followed by a number corresponding to their order of apparition on the chromosome (starting from *oriC*).

### Detection of expression using microarrays and Northern dotting

**Figure 3:**
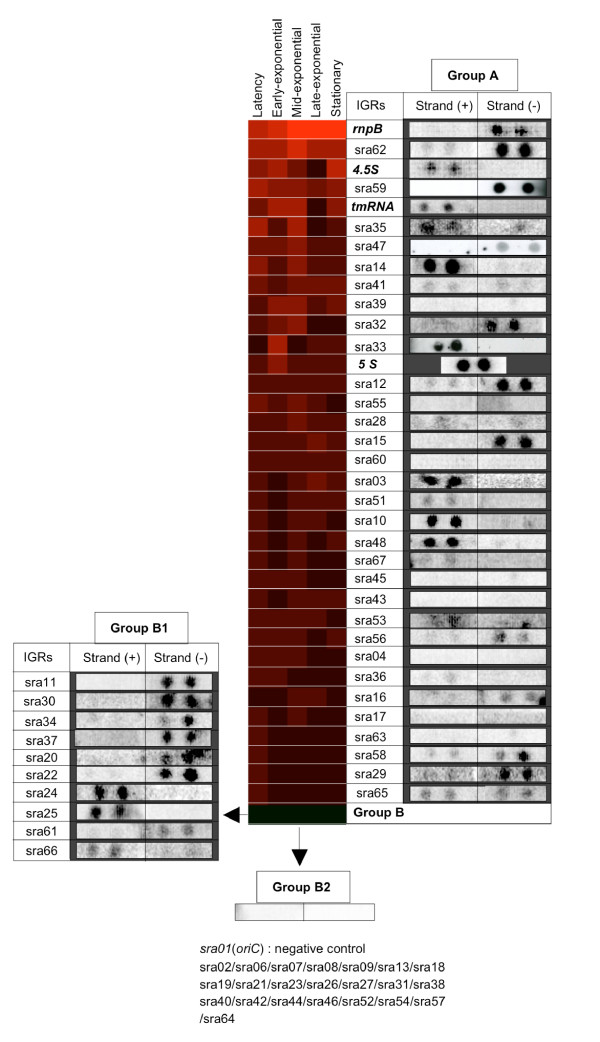
**Heat map and Northern dot expression analysis of *S. meliloti sra *genes**. Heat map visualizing the expression values for each *sra *gene at various stages of growth (in LB medium). Signal intensities are indicated as signal-to-noise SNR values (log2 scale), represented by a red panel. The position of each *sra *gene in the heat map is determined by its intensity. For the complementary Northern dot analysis, 10 μg of RNA, isolated from the most favourable expression condition, was spotted (in duplicate) onto a nylon membrane and hybridized with a radiolabelled specific probe [see Additional file 8].

Microarray hybridization was the first method used to test for transcription of the sRNA candidates; an IGR-dedicated array was used (see Materials and Methods). The initial goal of the time-course microarray experiments was to detect ncRNA gene transcripts in various conditions. We first monitored gene expression over time during growth in minimal (MV) and rich (LB) media. The average log_2 _SNR of the three known ncRNAs (*tmRNA*, *rnpB *and *4.5S*) and the ribosomal RNA 5S (included as a control) was very low (0.49) in MV medium, but was higher (4.13) in LB medium (see Additional file 5). We applied various stresses, as described in Materials and Methods, but detected no specific induction (not shown). We therefore used LB medium for expression analyses and the results are expressed as a heat map (Figure 3). We also checked the expression of all of the candidates by Northern dot blotting. Twenty-five candidates were found to give a strong hybridization signal (including *rnpB *and *4.5S *RNA genes) and nine gave weak but detectable signals. Among the dot blot-"positive" IGRs, ten did not show significant expression on microarrays (Fig. 3, group B1), perhaps due to insufficient fluorescent labelling (e.g. the RNA was too small or labelling was hampered by secondary folding). Twenty-three IGRs gave no transcription signal either in Northern dot blots or in microarrays (Fig. 3, group B2). These included *sra01*, which corresponds to the *S. meliloti *origin of replication (*oriC *or *smc04880*) and was used as an internal negative control. Indeed, various methods (including qPCR and blotting) have been used to demonstrate that there is no transcription at this site (not shown). Lastly, 11 IGRs giving a transcription signal on microarrays were not confirmed by Northern dot blotting; in these cases the signal may have originated from surrounding coding genes.

### Small RNA transcripts detected by Northern hybridization

Northern blotting was used to detect sRNAs. The same probes as for Northern dots were used but after hybridization of newly purified RNA from fresh cultures. For some candidates, probes were redrawn to target areas common to all considered subgroup-2 alpha-proteobacteria. *sra15 *was discarded because it appears to correspond to a pseudo *phe*-tRNA gene (not shown). This locus is possibly a vestige or a target of DNA insertion; indeed, a 500-kb symbiosis island has been shown to integrate into a phe-tRNA in the *M. loti *chromosome [57].

Four sRNA candidates (*sra51*, *sra37*, *sra47*, *sra59*) gave large transcripts (~1000 nt, not shown), inferred to be part of adjacent ORFs for three reasons: (i) they were larger than the intergenic region; (ii) they were large enough to encompass one of the flanking coding genes; and (iii) the strand detected corresponded to the orientation of at least one of the flanking genes. We did not pursue investigations to determine whether these 5'/3' UTRs encompass *cis*-regulatory RNA structures.

The signals detected for *sra48*, *sra58 *and *sra62 *were very strong and composed of multiple bands (data not shown). For *sra48 *and *sra58*, the explanation is sequence-specific hybridization of the probe to various transcripts due to small imperfect DNA repeats (*sm-2 *fragment in the case of *sra58 *and a previously undescribed repeat in *sra48*, data not shown). The *sra62 *candidate corresponds to a repeated region that coincides with the *fixT *loci, present in three copies in the genome of *S. meliloti *[40]. In the two first copies, *fixT *genes (*fixT1 *and *fixT2*) are co-located with fixK genes (fixK1 and fixK2). The third copy region of *fixT *gene (fixT3) encompasses *sra62*, which corresponds to a *fixK3 *pseudogene (not shown).

However, it cannot be concluded that the 23 candidates (group B2, Figure 3) for which no transcript was detected by microarray or Northern blot experiments correspond to false predictions: some of them may possibly be true sRNAs, but which are poorly or not expressed in the conditions tested.

### Analysis of the 17 newly identified small RNAs

**Figure 4:**
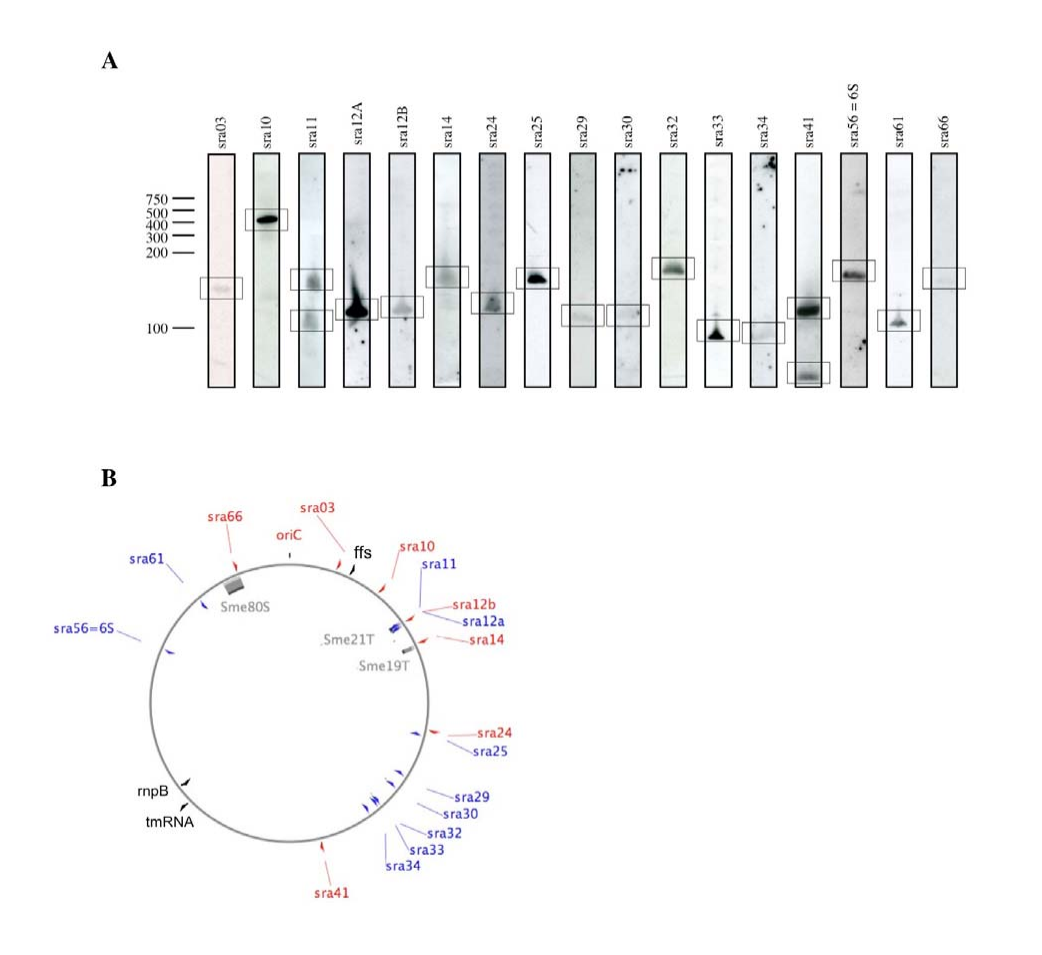
**Detection and location of chromosomal *S. meliloti *sRNAs**. A. Total RNA was extracted from cultures grown in LB (mid-exponential phase). Northern blots were hybridized with biotinylated DNA oligonucleotide probes [see Additional file 8] and exposed for various times (therefore the intensities of the signals do not correspond to the relative abundance of each sRNA). The positions of RNA size standards are shown on the left. B. Distribution of the *sra *genes along the *S. meliloti *chromosome. The origin of replication (*oriC *= sra01) and positions of *tmRNA*, *rnpB *and *4.5S *are also indicated. Blue and red arrows represent genes on the reverse or forward strands, respectively. Genomic islands are indicated with grey boxes (Sme21T, Sme19T and Sme80S).

We further investigated the 17 sRNA candidates that yielded signals with a suitable size in Northern blots (<300 nt, Figure 4A). We excluded (Table 2) *sra10*, that contains a possible 101 amino acid ORF and *sra29*, which is a probable leader for *rpsF *(S6 ribosomal protein gene) [58,59]; we also excluded *sra24 *that may encode a short peptide (38 aa long) rather rich in cysteine (10%). The 23 first aa (MALFFKPHCFLSLYCCLLSQRG...) correspond to a predicted transmembrane segment whereas 30% of the 14 other residues are positively charged amino acids. This biochemical configuration is found in small molecular peptide ion channels, such as defensins, involved in host-defences.

**Table 2:** Features and annotations of the 3 new small RNAs of *S. meliloti *potentially encoding proteins and peptides

	sra10	sra24	sra29
Flanking genes	rpsT-dnaA	Y00125-027	rpsF-Y00569
Orientation	> > >	< > >	< < >
Estimated size(s) (nt)	383	104	110
R. etli CFN 42	•	-	•
R. leguminosarum	•	-	•
A. tumefaciens C58/C	•	-	•
A. tumefaciens C58/W	•	-	•
M. loti MAFF	-	-	•
B. suis	-	-	•
B. melitensis	-	-	•
B. abortus	-	-	•
			
Possible functions	ORF	Peptide	5' UTR

Of the 14 remaining RNAs, six correspond to *S. meliloti*-specific sRNAs (called orphan sRNAs, Table 3). All except *sra61 *lie within a genomic island [60] (*sra14 *in Smc19T; *sra11*, *sra12a *and *sra12b *in Smc21T; *sra66 *maps at the 5' end of Sme80s, just downstream from the insertion tRNA^ser ^gene *smc03779*). All these sRNAs are absent from *Agrobacterium *and other *Rhizobium *species, consistent with the absence of the three genomic islands from these related genomes. The "coding potential" of *sra11*, *sra12a*, *sra12b *and *sra14 *was assessed and only *sra66 *could translate a small non-conserved 16 aa peptide. There may be a relationship between their biological functions and their presence in genomic islands as bacterial islands are often related to eukaryotic host cell colonization (virulence or symbiosis). Indeed bacterial sRNAs were recently detected in the pathogenicity island of *Staphylococcus *[51] and in *Salmonella *(InvR [36]). Multiple 5' and 3' RACE mapping of these candidates was unsuccessful, possibly due to insufficient cDNA or because they are too small for satisfactory cloning. With no available orthologous sequences, determination of extremities through alignment and structure prediction based on covariations are not possible. The functions of these new non-coding genes are thus unknown. However, many small RNAs in bacteria act as post-transcritptional regulators via base-pairing action, so we used TargetRNA [61] to assess their capacity to act as antisense RNAs. No significant putative target was found for *sra11*, *sra12a*, *sra14 *or *sra61*, but *sra12b *and *sra66 *match with mRNA targets (see Additional file 12). One interesting TargetRNA prediction was the interaction between *sra66 *and a tolR-exbD-like gene (*smc03957*). TolR-ExbD proteins are membrane-bound transport proteins essential for ferric ion uptake in bacteria [62], and small RNAs modulating the free intracellular iron pool have indeed been identified in other bacteria (e.g *RyhB*, [63]).

**Table 3:** Features and annotations of the 6 new orphan (*S. meliloti*-specific) sRNAs of *S. meliloti*

	sra11	sra12a	sra12b	sra14	sra61	sra66
Flanking genes	Y02201-2189	Y02151-50	Y02151-50	Y02287-2205	Y03108-3107	Y003769-79
Orientation	> < <	< < >	< > >	< > <	> < <	< > <
Estimated size(s) (nt)	150 and 90	104	104	120	89	144
Possible functions	UD	UD	Antisense	UD	UD	Antisense

UD : undetermined

In *E. coli*, sRNA genes were considered to be conserved if the alignment had an E-value lower than 0.001 [64]. On this basis, the sequences of the eight remaining expressed *S. meliloti *sra genes are highly conserved in related alpha-proteobacteria (e-value < 10^-10^; Table 4). *sra56 *is even an analog of *Escherichia coli*'s 6S RNA (*SsrS*) that has recently been corroborated by RFAM. *sra56 *may thus be considered as a fortuitous positive control, validating our experimental approach. All these conserved *sra *genes we describe were then subjected to transcriptional element analysis, structure prediction using covariation, and potential mRNA target prediction.

**Table 4:** Features and annotations of the 8 new Alpha-proteobacterial sRNAs of *S. meliloti*

	sra03	sra25	sra30	sra32	sra33	sra34	sra41	sra56
Flanking genes	polA-Y02851	Y00034-96	Y01755-aspS	Y01933-proS	celR2-rpmG	tufB-Y01325	Y01226-25	Y03975-76
Orientation	< < >	> > <	< < >	< < >	> > <	> < >	< < >	> < <
Estimated size(s) (nt)	120	144	104	133	76	76	106 & 68	127
*R. etli CFN 42*	•	•	-	•	•	-	•	•
*R. leguminosarum*	•	•	-	•	•	-	•	•
*A. tumefaciens C58/C*	•	•	-	•	•	-	•	•
*A. tumefaciens C58/W*	•	-	-	•	•	-	•	•
*M. loti MAFF*	-	-	-	•	•	-	•	•
*B. suis*	-	-	-	-	-	-	-	•
*B. melitensis*	-	-	-	-	-	-	-	•
*B. abortus*	-	-	-	-	-	-	-	•
*Possible functions*	UD	UD	UD	Antisense	Antisense	Antisense	Antisense	6S

UD : undetermined

Dot (•) represents e-values > 10e^-4^

### Genomic synteny for alpha-proteobacteria *sra *genes

*S. meliloti *chromosomal sRNA-encoding genes have a slight distribution preference, 70% of them being on the left replicore, but no preference was observed concerning the leading or the lagging strand (Figure 4B). The conservation of gene adjacency (synteny) may be associated with functional relationships (e.g. *gcvB *and *ssrA *are functionally associated to their conserved adjacent genes, respectively *gcvA *and *smpB*) [35,65]. Therefore, we assessed the conservation of neighbouring genes for the eleven alpha-proteobacterial *sra*, including *tmRNA*, *rnpB *and *4.5S *(see Additional files 10 and 11). We found only three (*4.5S*, *tmRNA *and *sra41*) where both flanking genes were conserved and four with one conserved flanking gene (including *rnpB*). Three display no synteny (including *sra56 = 6S*). However, our observations suggest that in alpha-proteobacteria, functional association between sRNAs and conserved adjacent genes is uncertain, as even *smpB *is not linked to tmRNA (separated by 1,1 Mb).

### Conserved *sra *gene transcriptional signals

The computational identification of promoter sequences is difficult because signals are weak [4]. Consensus binding sites have been proposed for the *S. meliloti *σ^70 ^and σ^54^-dependent promoters (CTTAGAC-n17-CTATAT [66] and TGGCACG-n4-TTGCW [67], respectively). Using relaxed regular expressions (i.e. pattern matching), we scanned the 5' UTR region of each *sra *gene to detect any such consensus (max. 4 mismatches). In the same way than for the orphan candidates, the end-mapping experiments remained unsuccessful for all new candidates but *sra32 *(5' end-AAACAGGCAGGAA and 3' end-CTTGTTTTTTT), thus the exact sites of the extremities of the *sra *genes are unknown. However, sequence alignment between several alpha-proteobacteria was informative. As for ORFS, no significant nucleotide identities were apparent in the 5' and 3' regions (not shown). Thus, the start and stop of the alignment and the sizes deduced from electrophoretic motility were used to define the ends of each sra gene. We only detected consensus σ^70^-promoters in the ubiquitous ncRNAs (*ffs*, *tmRNA*, *rnpB *and *6S*) and the 5'-UTR mapped region of *sra32 *(Table 5). Even with a larger spacer between the -35 and -10 boxes (17 to 20), no consensus sequence was detected in the other *sra *genes. Similarly, only the *ffs *5'-UTR matches a possible σ^54 ^binding site. However, *S. meliloti *contains a large number of predicted sigma-factors (ca. 16), it is therefore possible that *sra *genes are dependent on them. Unfortunately, Melina-based [68] motif extraction from the promoter regions of these genes was inconclusive and did not show any consensus.

**Table 5:** Promoter detection in conserved *S. meliloti sra *genes

	σ70-dependent [68]	σ54-dependent [68]
*tmRNA*	tTTtct-n17-TATATg [41]	-
*sra05-ffs*	CTTgca-n17-cgATAT	tTGGCtt-n4-TTGCT
*sra50-rnpB*	CTctGgC-n17-tTtaAT	-
*sra56-6S*	CTgGtG-n17-CTATAT	-
*sra03*	-	-
*sra25*	-	-
*sra32*	gaTGAC-n17-CTA.AT	-
*sra33*	-	-
*sra41*	-	-

We also looked for potential classical L-shaped terminators (stable stem-loops followed by U-stretches) at the alignment-deduced 3' end of each *sra*. As most terminators in GC-rich bacteria have no long consecutive U-stretches [69], we also looked for I-shaped (stable stem-loop with no U-stretch) and V-shaped (two consecutive hairpins [69]) terminators (Table 6). Terminator structures could be predicted for all conserved sra genes, except *sra30 *and *sra34*, for which the alignments are too short for the ends to be accurately mapped. All three shapes were found, I-shapes being the most common. This supports the proposal that "orphan" terminators in IGRs may indicate the presence of an *sra *gene, although because of the high GC content of the *S. meliloti *genome, stable stem-loops are frequent and consequently not very informative.

**Table 6:** Terminator prediction for *S. meliloti sra *genes

	Sequences or references	Shape type [69]
*tmRNA*	P6 and P7 stem-loops [41]	V-shaped
*sra05-ffs*	GGCACGG-n4-TCGTGCC-agcct	I-shaped
*sra50-rnpB*	GTTTCGTCTC-n4-GAGGCGGAGC-catac	I-shaped

*sra56-6S*	CGGTC-n6-GATCG-cactt	I-shaped
*sra03*	CGGCATT-n5-AGTGCCG-ctcga	I-shaped
*sra25*	GGGCCGCC-n3-GGGCGGCCC-tttt	L-shaped
*sra32*	TB8 (cf figure 5)	I-shaped
*sra33*	TG_4_TCGG-n4-CTGGCCTCG *et *T2GCGG-n10-CTGCAA-	V-shaped
*sra41*	AGGGCCcAAG-n5-CTTGtGGCCCT-cttttttt	L-shaped

### Conserved *sra *genes predicted structure and mRNA targets

The structures of non-coding RNAs are important for their function. Consequently, we made a conserved secondary structure prediction for all alpha-proteobacteria *sra *genes (except *sra30 *and *sra34 *because of too short alignments).

We first compared the 4.5S and 6S Alifold [70,71] and RNAz [72], outputs with the equivalent RFAM structure (Additional file 13). The two tools give different structures for 4.5S, the Alifold prediction being closer to that of RFAM than the RNAz prediction. However, bulge A, the minimal site required for binding to EF-G [73], is similarly folded in the three models. The three predicted structures of *sra56 *(6S) are similar, 6S being folded as a largely double-stranded RNA with a single-stranded central bulge. However, the 3' side of the central bulge can form a stable stem-loop in alpha-proteobacteria, as previously described [74].

The predicted structure of *sra03 *is well-conserved, composed of three long hairpins: the central one, ending with a 15 nt loop, is strictly conserved, and the 3' and 5' stem-loop structures present co-varying nucleotides. Analogous design (three stem-loops) was found for the *E. coli *sRNAs *drsA*, *micC *and *qrr *[46]. No significant target mRNA was found using the full RNA but the 15-nt loop presents complementarity with the 5'-UTR of the *smc03977-smc03976 *operon (not shown). These two genes have paralogs, mostly in alpha-proteobacteria genomes. The function of Smc03977 is unknown but Smc03976 belongs to the ZapA cell division family of proteins [75]. ZapA binds the *ftsZ *cell division gene, which has been reported to undergo antisense inhibition in *E. coli *[22], so *sra03 *is possibly implicated in cell division regulation.

*sra25 *was only identified in Rhizobia (*meliloti*, *etli *and *leguminosarum*), however with remarkable primary sequence identity. Its predicted secondary structure is also highly conserved, composed of a long stem with one central bulge and a small hairpin. The 3' end structure was predicted to act as a stabilization terminator. A possible pairing with the 5' UTR of a gene encoding a putative membrane protease protein (Smc04020) was proposed by TargetRNA, although with low significance (*p-value *> 10^-3^). This protein resembles the *E. coli *HflKC protein, known to interact with the cell division protease FtsH [76]. As for *sra03*, we propose that *sra25 *may have a role in the *S. meliloti *cell cycle.

Only Alifold proposed a consensus structure for *sra33*: it resembles other short bacterial sRNAs including RyeE [46]. Predictions for *sra33 *showed strong pairing ability with the 5' ends of *smc00899 *and of *rkpJ*. The first gene is organized in an operon structure with *smc00900 *(encoding a PilT-like toxin), defining a post-segregational killing toxin-antitoxin (TA) system. This type of mechanism, involving sRNAs in the inhibition of TA systems, has been extensively described in *E. coli *[77]. Finding such a mechanism in *S. meliloti *is interesting as we recently detected approximately 53 TA loci (accounting for 95 genes) in the complete genome sequence [78].

**Figure 5:**
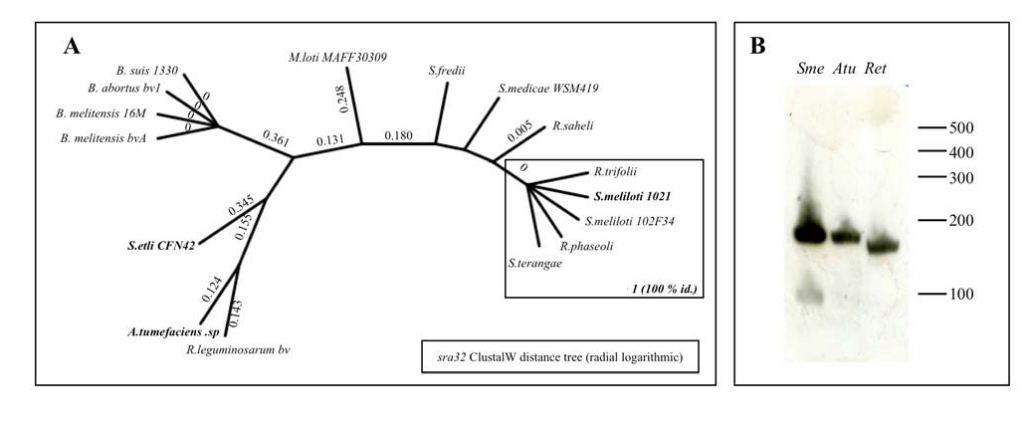
**Characterization of *sra32***. A. Distance tree of *sra32 *using the distance/NJ method (clustalW [90]). Numbers at the nodes represent bootstrap proportions (BP) of 1000 replicates. B. In northern blots, a signal for the predicted *sra32 *was detected corresponding to lengths of 144 and 106 nucleotides in total RNA from *S. meliloti *1021 and to 140 and 132 nt in that from *Agrobacterium tumefaciens *and *Rhizobium etli*, respectively.

*sra32 *is the only small RNA predicted both by sRNApredict2 and ACT analysis. For this gene, hybridization was tested by Northern blot and a signal was obtained for two of the most distant members: *A. tumefaciens *(140 nt) and *R. elti *(132 nt) (Figure 5A and 5B). The secondary structures of *sra32 *were predicted by both Alifold and RNAz; 59% of the nucleotides can pair in seven conserved regions, those at the 3' and 5' ends (TB1 and TB7) possibly forming stabilization stem-loops. Only the primary sequence of TB3 is conserved, all other structures being supported by covariations. The search for potential mRNA targets yielded significant predictions for *fliM *(flagellar motor switch) and *smc01800 *(cytochrome C oxidase). The same sequence of *sra32 *(nt 60 to 95, TB4 to TB6) is predicted to pair with the 5' leader region of both mRNA targets. Discovery of a putative flagella antisense RNA in *S. meliloti *and related bacteria is interesting. Indeed, all these bacteria interact with plant or mammal cells and flagella are cell-surface components required for eukaryotic-prokaryotic cells interactions [37].

Finally, structure and target prediction of *sra41 *needed preliminary analysis as (i) two signals were detected by Northern hybridization (a major ~106 nt band and a minor ~68 nt species) and (ii) *sra41 *is present in three imperfect copies, two in tandem in the same chromosomal IGR and the third, with a more divergent primary sequence, maps in pSymA. Additionally, *sra41 *is well-conserved in alpha-proteobacteria and is present in two to three copies, on both chromosomes and (mega-)plasmids except in *Agrobacterium *(chromosomal loci only). Translatable small ORFs are predicted within some *sra41 *candidates (see Additional file 6) whereas others are devoid of complete coding frames. A similar conserved structure was proposed for the three *S. meliloti *copies, composed of three stem-loops as described for *sra03*. Target mRNAs were predicted separately for each of the three *sra41 *copies in *S. meliloti*. The only predicted target for the first chromosomal copy is *smc02392*, which encodes a hypothetical protein with a Sel1 repeat-containing domain. The second copy is predicted to interact with two mRNAs encoding Smc00317, a transmembrane protein with homologies to efflux carrier proteins, and Smc01118, a glucoprotease-like protein possibly involved in chaperoning processes. No significant target was predicted for the pSymA copy.

## Conclusion

We used computational comparative genomic screening to search for small RNA genes in *S. meliloti *and related alpha-proteobacteria, as very few sRNAs had been identified in this phylum [46]. From a list of 64 *S. meliloti *candidate IGRs (excluding *tmRNA*, *4.5S *and *rnpB*), we show that 17 encode small RNAs (14 non-coding RNAs, two small mRNAs and a 5' leader region). This work constitutes a significant advance in small RNAs studies in alpha-proteobacteria.

A possible antisense function was suggested for 57% of the *sra *genes, although these predictions remain based on TargetRNA estimations. To validate these antisense activities, further in vitro validation of predicted target genes will be necessary, even if TargetRNA's e-value was set to a highly significant threshold (see material and methods section). It is however interesting to notice that the functions of the predicted mRNA targets are various, including roles in transport, membranes and toxin-antitoxin systems. However, antisense RNAs may act on transcription termination, translation, mRNA degradation and can be activators and/or repressors. As a consequence, accurate *in silico *prediction of *sra *functions is difficult. The specific physiological roles of these newly discovered genes in alpha-proteobacteria regulatory pathways can only be determined by biological investigation. The initial regulation of each *sra *gene, i.e. the precise signalling conditions that trigger their expression should be analyzed, as this may give us clues about their roles. Monitoring the expression of each *sra *under various conditions would require numerous experiments, as many parameters can be changed alone or simultaneously to simulate oxidative, heat, cold, and osmotic stresses as well as nutrient and metal starvation. In *E. coli*, Hfq generally facilitates the pairing of ncRNAs [79]. Therefore the influence of the *S. meliloti hfq *homolog (*nrfA *or *smc01048*) in the *sra*-target mRNAs hybrid formation could be analyzed by constructing a mutant. In parallel, screening should be extended to additional replicons (megaplasmids and plasmids): we show that sequence conservation in closely related bacteria IGRs can indicate the presence of putative small *sra *genes.

## Methods

### Bacterial growth

In all experiments, *S. meliloti *strain 1021 (streptomycin resistant, Str^R^) was grown at 30°C in the presence of 25 μg.ml^-1 ^of streptomycin. Bacteria were initially grown to the stationary phase in LB (Luria-Bertani) medium, collected, washed and then resuspended in MV (Vincent minimal medium, 1970 [80]) or LB medium. Stresses were applied in MV at mid-exponential phase (OD600 nm = 0.4) as follows: 10 minutes with a sub-lethal dose of hydrogen peroxide (10 mM); salt shock (0,4 M NaCl), pH 5 and pH 9 generated with 10 N HCl or NaOH were also applied during 10 minutes; cold (10°C) and heat shocks (40°C) lasted 15 minutes. The other alpha-proteobacteria used in Northern blot and sequencing experiments are listed in Additional file 7.

### RNA purification and DNase treatment

Cells were harvested by centrifugation (5 min, 2600 × g, room temperature) and immediately frozen at -80°C. These pellets were subsequently re-suspended in 200 μl of TE buffer (10 mM Tris-HCl pH8 – 1 mM EDTA) containing lysozyme (1 mg.ml^-1^) and total RNA was extracted using the RNeasy^® ^mini kit (Qiagen™) and treated with RQ1 Rnase free DNase (1 U.μg^-1^, GE Healthcare) in the presence of 6 mM of MgCl_2 _for 20 min at 37°C.

### 5' and 3' RACE mapping

RNAs shorter than 300 nt were eluted from a 2.5% high-resolution agarose gel (Sigma) in 0.5 × TBE buffer [81] and used to build a cDNA library using the MessageAmpII-Bacteria kit (Ambion). The RNAs were 3' end-polyadenylated and first strands were synthesized using oligo-T7-dT (AATACGACTCACTATAGGGCGAA dT25) then treated overnight at 37°C with terminal deoxynucleotidyl transferase in the presence of 1 mM of dTTP. The second strands were obtained with a 5'-primer (GGAATACTAGTGACACCAGACAAGTTG dA15). PCR was carried out at 55°C using 1 μl of the cDNAs and 4 pmoles of the appropriate specific primers (see Additional file 8): one corresponding to the targeted gene and the other specific to either the 5' or 3' tagged ends. The products obtained were inserted into pGEM-T (Promega) and sequenced using the BigDye terminator v3 protocol (Applied Biosystems). RNA genes of interest were amplified by PCR with a 50°C to 40°C touch-down program, using the 5' and 3' end primers designed after mapping, from 12 genomic DNA preparations from several Rhizobia (see Additional file 8). The resulting products were sequenced using the protocol described above and analyzed *in silico *for co-variation and conserved secondary structure.

### Northern dot analysis

DNase-treated, *S. meliloti *total RNA (10 μg per dot) was denatured (by incubating for 10 min at 70°C then chilling on ice) and spotted under vacuum onto a nylon membrane (Zeta Probe) using Bio-Dot (Bio-rad). These dot membranes were baked for 30 min at 80°C, pre-hybridized, hybridized and washed according to Ambion's instructions (Oligoprobes), and hybridization was visualized on InstantImager (Packard). 20-mer oligonucleotides (see Additional File 3) were 5' P-end labelled with [gamma^32^P]ATP. For each candidate, two oligonucleotide probes (one per strand) were selected to identify the strand of transcription. Aliquots of 62 pmoles of each oligonucleotide probe were incubated for 1 hr at 37°C with 1 μl of T4 polynucleotide kinase, 2.5 μl of 10 × kinase buffer (Ambion) and 2 μl of ^32^P-ATP [5,550 KBq (222,000 KBq.mmol^-1^)]. The radiolabelled probes were then purified through MicroSpin G25 columns (GE Healthcare). The efficiency of probe labelling was monitored in a liquid scintillation analyser (1600 TR Packard).

### Northern blot analysis

*S. meliloti *was grown in LB medium to mid-exponential phase (OD_600 nm_= 0.8). Total RNA (10 μg) was extracted, denatured, and subjected to electrophoresis on an 8% acrylamide/bis acrylamide (19:1) denaturing gel (8 M urea) in 0.5 × TBE buffer [81]. The RNA was then transferred to a nylon membrane (Ambion) in the same buffer and the membrane was baked for 30 min at 80°C. Biotinylated oligoprobes were used for hybridization and bound probes were detected according to Ambion's instructions (BrightStar^® ^BioDetect™).

### Construction of the DNA microarrays

A PCR-based microarray was designed (see Additional file 9) with primer3-designated oligonucleotides derived from the 67 IGRs (intergenic empty regions) identified by screening (see Additional File 4). Seventy bp were excluded at the 5' and 3' ends of each IGR to avoid expression contamination by adjacent genes. The IGRs with lengths >550 nt were amplified as more than one PCR fragment. Each PCR involved 200 ng of *S. meliloti *1021 genomic DNA, 1.25 units of *Taq*DNA polymerase (Promega) and 4 pmol of each oligonucleotide in a final volume of 50 ml for 40 cycles (Peltier Thermal Cycler, MJ Research Inc). The PCR buffer contained 5 μl MgCl_2 _(25 mM), 1 × Taq buffer (Promega) and 4.3 μl dNTPs. The length and quality of each PCR product was assessed by electrophoresis on 2% agarose TBE (Tris-Borate EDTA 0.5 × final) gels. The IGRs-PCR probes were dried, resuspended in 30 μl betaine, 1.5 M-3 × SSC and spotted in triplicate onto GAPS II™-coated glass slides (Corning) through robotic arraying (Microgrid II, BioRobotics).

### Microarray hybridization, data acquisition and statistical analysis

The labelled cDNA was resuspended in hybridization buffer (3 × SSC/0.1% SDS/50% formamide) and cohybridized with 10 μg of salmon sperm DNA to the microarray glass slides overnight at 55°C. The slides were washed for 5 min at 55°C in wash buffer (2 × SSC/0.1% SDS) then 2 min in high-stringency buffer (0.2 × SSC/0.1% SDS, twice), 2 min in 0.2 × SSC (twice) and 2 min in 0.1 × SSC, and dried by centrifugation (3 min, 210 g). Hybridized microarray slides were scanned (GenePix4000, Axon Instruments, Inc.) with independent excitation of the fluorophores Cy5 and Cy3 at 10-μm resolution. Our microarray time series experiment was planned with a dye-swap design; Dabney & Storey recently showed that a simple average dye-swap removes dye bias without affecting the biological signal and preserves the ordering of true expression means [82]. To determine if ncRNA candidates showed detectable expression as assessed by microarray analysis, we used log_2 _SNR (signal to noise ratios), the "noise" value being the average intensity of spots containing spotting buffer. All genes with log_2 _SNR lower than 2 (a signal less than 4-fold greater than the technological noise) were considered as untranscribed under the conditions tested and their value changed to 0 (filtering). The resulting expression profiles are illustrated as a heat map.

### Bioinformatics

Intergenic regions (IGRs) were determined as genome areas with no gene annotation on either of the two strands. The *S. meliloti *database used [83] includes original annotations of all ORFs, as well as tRNA, rRNA and repetitive elements. The Artemis Comparison Tool (ACT [84]) from the Sanger Centre [85] was used for whole genome comparisons of IGRs between the *S. meliloti *chromosome, as the reference, and other alpha-proteobacteria genomes: *Agrobacterium tumefaciens *strain C58 Cereon, *Rhizobium etli *CFN42, *Rhizobium leguminosarum *bv. *vicae *3841 and *Mesorhizobium loti *MAFF303099.

ISI [54] was used taking the initial embl record file corresponding to the *S. meliloti *chromosome (AL591688) as an input, and applying an IGR length threshold of 7 bp. Parameter 'w' in BLAST was set to 7 in order to refine word detection.

In order to produce the input alignment file for QRNA v.2.0.3.c [7], the 61 ncRNAs annotated in the *E. coli *K12 Refseq file (NC_000913) were extracted, and compared to the chromosomal IGRs of *S. meliloti *thanks to WU BLAST 2.0, with various e-value thresholds (10^-5^, 10^-2^, 0.1 and default: 10). QRNA was then run with scanning window set to 100 nt.

sRNAPredict2 [18] was tested using the *S. meliloti *chromosome ORF list (NC_003047.ptt) from the Refseq repository [86]. The ".coords" TIGR file is unavailable for *S. meliloti*. The t/rRNA database was compiled from data in the TIGR_CMR RNA list [87] and from the *S. meliloti *sequencing consortium website [83]. The terminator database was derived from TransTerm [88,89]. The *S. meliloti *sRNA training set was generated from data available at the corresponding RFAM database page [46]. Two reference genomes were used: *Agrobacterium tumefaciens *str. C58 and *Rhizobium etli *CFN 42. Consequently, two blast output files were generated, where *S. meliloti *intergenic regions were compared to the cited genomes using WU BLAST 2.0 with same parameters as Livny [18] (E = 10e^-5^, V = 10000 et B = 10000). Finally, databases of regions of predicted conserved secondary structure were produced using QRNA, again with parameters set to the same values as in [18]. No input files corresponding to RNAMotif search results or to promoter/transcription binding sites were provided to sRNAPredict.

TargetRNA [61] was used (default parameters) to identify *sra *candidates' mRNA targets. Potential base pair binding interactions were considered significant only if their *P*-value fell below 0.001. Nucleotide sequences were analyzed by ClustalW [90] and resulting multiple alignments were used in ALIFOLD [71] to model RNA secondary structures. Similar predictions were realized using the RNAz program [72].

## Authors' contributions

VU and AC conceived the design and use of the microarrays and performed the isolation and blotting of the RNAs. AC performed 5'-3' RACE and sequencing of *sra32 *candidates in Rhizobiales. ES and FBH performed the bioinformatic analyses and IGRs annotation. FBH designed and coordinated all aspects of the project. All authors read and approved the final manuscript.

## Supplementary Material

Additional file 1 Phylogenetic maximum likelihood trees of alpha-proteobacteria *ffs*, *rnpB *and *tmRNA *sequences. The data provided shows the phylogenetic maximum likelihood trees in subgroup-2 alpha-proteobacteria of the canonical *tmRNA*, *rnpB *and *ffs *small RNAs. Click here for file

Additional file 2 BlastN detection of *E. coli *small genes in *S. meliloti *genome. The data provided describes the results of homology searches for *E. coli *small RNAs in the *S. meliloti *genome. Click here for file

Additional file 3 sRNAPredict outputs. The data provided presents the results obtained with sRNAPredict on *S. meliloti*'s chromosome. Click here for file

Additional file 10 Short (FASTA) alignment and corresponding synteny results. The data provided shows the results of the search for small regions of homology between alpha-proteobacteria. Click here for file

Additional file 11 Artemis Comparison Tool (ACT) screenshots alignment and corresponding synteny results. The data provided presents the results of the ACT comparisons. Click here for file

Additional file 4 Intergenic regions included in this study. The data provided lists the intergenic regions selected (sra candidates) for *in vivo *validation and their features. Click here for file

Additional file 5 Normalized expression levels (log_2_SNR) of IGRs candidates. The data provided compiles the normalized expression levels for the candidates. Click here for file

Additional file 12 TargetRNA results. The data provided shows the results of the target analysis for each valid sra gene. Click here for file

Additional file 13 Alifold and RNAz secondary predictions. The data provided presents the alternative structures predicted for sra genes depending on the tools used. Click here for file

Additional file 6 sra41 multiple loci in *S. meliloti *and related alpha-proteobacteria. The data provided features the multiple loci of sra41 in subgroup-2 alpha-proteobacteria. Click here for file

Additional file 7 Strains used in this study.The data provided references the different bacteria used in this study. Click here for file

Additional file 8 Oligonucleotides used in RACE and Northern assays. The data provided presents the oligonucleotide sequences used in RACE and Northern assays. Click here for file

Additional file 9 Oligonucleotides used in microarray design. The data provided presents the oligonucleotide sequences used in the microarray design. Click here for file
