# Supplementary material for: Identification of chromosomal alpha-proteobacterial small RNAs by comparative genome analysis and detection in Sinorhizobium meliloti strain 1021
Source: BMC Genomics. 2007 Dec 19;8:467. doi: 10.1186/1471-2164-8-467 (PMC2245857; doi:10.1186/1471-2164-8-467)
Supplement: Additional file 1 — Phylogenetic maximum likelihood trees of alpha-proteobacteria ffs, rnpB and tmRNA sequences. The data provided shows the phylogenetic maximum likelihood trees in subgroup-2 alpha-proteobacteria of the canonical tmRNA, rnpB and ffs small RNAs. [file 1471-2164-8-467-S1.pdf]

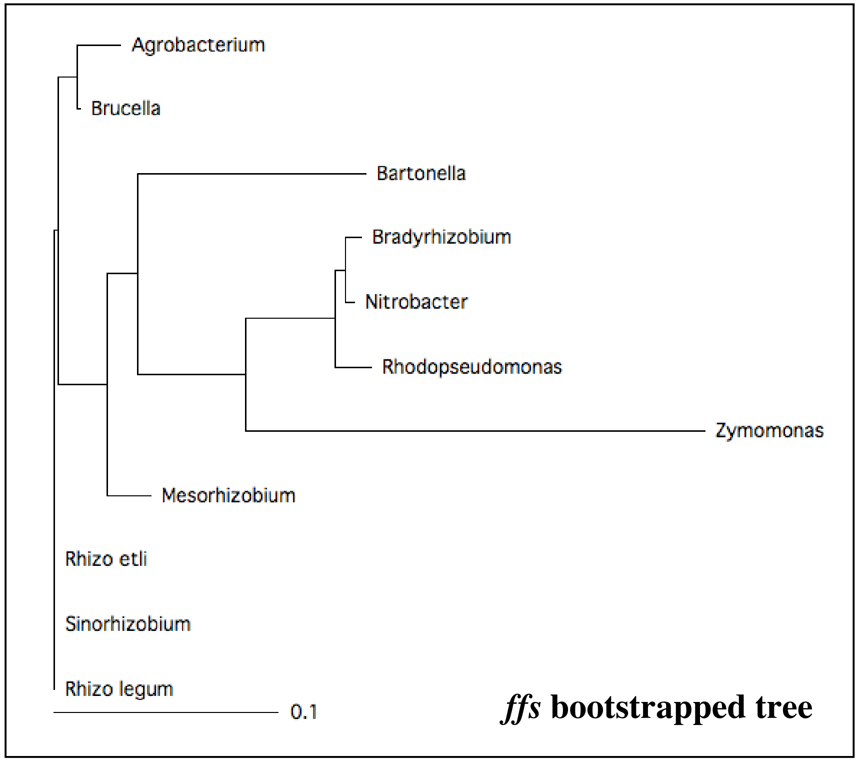

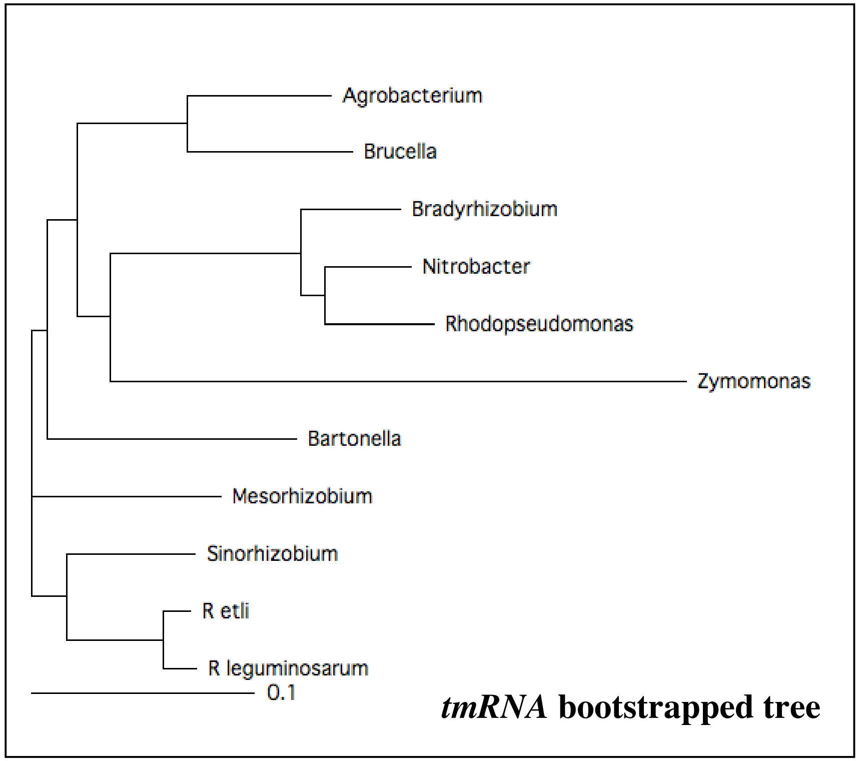

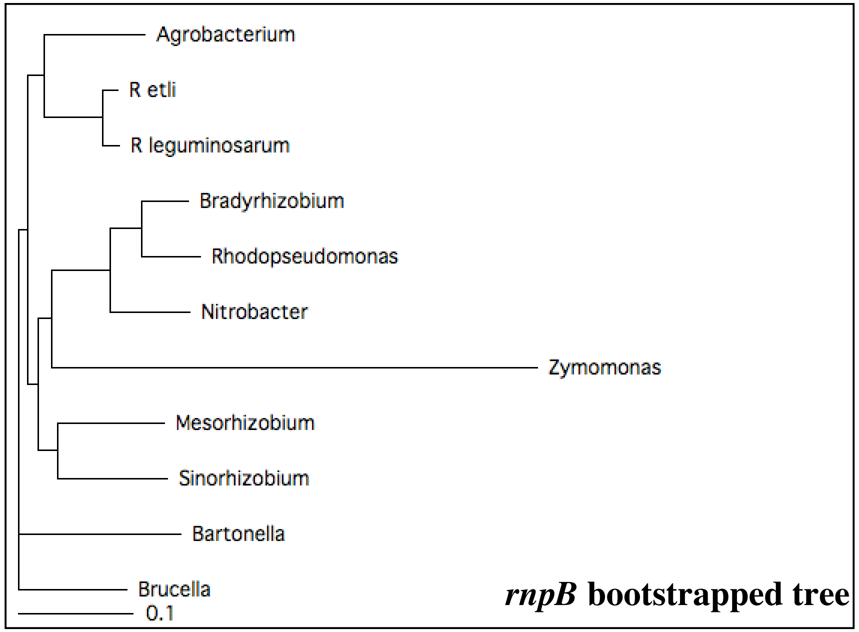

Phylogenetic maximum likehood trees based on *ffs*, *rnpB* and *tmRNA* in the subgroup-2 alphaproteobacteria.

These unrooted tree derived from the comparison of the complete predicted sequences of *ffs*, *tmRNA* and *rnpB* small RNAs [ref] from closely related alpha-proteobacteria. The analysis used Neighbourg joining method. Bootstrap values (1000).
