# Supplementary material for: Identification of chromosomal alpha-proteobacterial small RNAs by comparative genome analysis and detection in Sinorhizobium meliloti strain 1021
Source: BMC Genomics. 2007 Dec 19;8:467. doi: 10.1186/1471-2164-8-467 (PMC2245857; doi:10.1186/1471-2164-8-467)
Supplement: Additional file 2 — BlastN detection of E. coli small genes in S. meliloti genome. The data provided describes the results of homology searches for E. coli small RNAs in the S. meliloti genome. [file 1471-2164-8-467-S2.pdf]

Gene in *E. coli*	Hit in *S. meliloti* (e-value < 1)	Phylogenic repartition
**<u>RNA genes</u>**		
4.5S-ffs	e = 0.74 , Score = 141	ubiquitous
rnpB	e = 2.2e-^{08}, Score = 302	ubiquitous
6S-ssr	no significant hit	ubiquitous
tmRNA	no significant hit	ubiquitous
**<u>small RNA genes</u>**		
QUAD	no significant hit	*Shigella*, *E. coli, Salmonella*
SraE_RygA_RygB	no significant hit	*Shigella*, *E. coli, Salmonella, Yersinia*
DicF	no significant hit	*Shigella*, *E. coli, Salmonella, Bacillus*
SgrS	no significant hit	*Shigella*, *E. coli, Salmonella*
RydC	no significant hit	
sroH	hit in ORF (*sma02053-mocE*) e = 0.29, Score = 136	*Escherichia coli*
sroE/D/C	no significant hit	*Shigella*, *E. coli, Salmonella*
sroB	no significant hit	*Shigella*, *E. coli, Salmonella, Yersinia*
tke1	no significant hit	*Shigella*, *E. coli, Salmonella, Yersinia*
t44	no significant hit	*Shigella*, *E. coli, Salmonella, Yersinia, Photorhabdus, Pseudomonas, Coxiella*
ryfA	no significant hit	*Shigella*, *E. coli, Salmonella*
IS128	no significant hit	*Shigella flexneri*, *Escherichia coli*
IS102	no significant hit	*Shigella flexneri*, *Escherichia coli*
GadY	no significant hit	*Shigella flexneri*, *Escherichia coli*
MicC	no significant hit	*Shigella*, *E. coli, Salmonella, Clostridium*
C0343	no significant hit	*Shigella*, *E. coli, Salmonella*
C0299	no significant hit	*Shigella flexneri*, *Escherichia coli*
rydB	no significant hit	*Shigella*, *E. coli, Salmonella*
C0719	no significant hit	*Shigella flexneri*, *Escherichia coli*
C0465	no significant hit	*Shigella flexneri*, *Escherichia coli*
IS061	no significant hit	*Shigella flexneri*, *Escherichia coli*

RyeE/B	no significant hit	*Shigella, E. coli, Salmonella, Yersinia, Erwinia, Photorhabdus*
RybB	no significant hit	*Shigella, E. coli, Salmonella, Yersinia, Erwinia, Photorhabdus*
SraC_RyeA	no significant hit	*Shigella, E. coli, Salmonella, Yersinia, Erwinia, Photorhabdus*
CsrC	no significant hit	*Shigella, E. coli, Salmonella, Yersinia*
SraJ	no significant hit	*Shigella, E. coli, Salmonella, Yersinia, Photorhabdus*
SraG/H	no significant hit	*Shigella, E. coli, Salmonella, Yersinia, Erwinia, Photorhabdus*
SraD	no significant hit	*Shigella, E. coli, Salmonella, Yersinia, Erwinia, Serratia*
SraB	no significant hit	*Shigella, E. coli, Salmonella*
RyhB	no significant hit	*Shigella, E. coli, Salmonella, Yersinia, Erwinia, Photorhabdus*
		Photobacterium, Pectobacterium, Vibrio
Rne5	no significant hit	*Shigella, E. coli, Salmonella, Yersinia, Erwinia*
OxyS	no significant hit	*Shigella, E. coli, Salmonella*
MicF	no significant hit	
GcvB	no significant hit	*Shigella, E. coli, Salmonella, Yersinia, Erwinia, Photorhabdus*
		Photobacterium, Haemophilus, Vibrio, Pasteurella, Mannheimia
Spot_42	no significant hit	*Shigella, E. coli, Salmonella, Yersinia, Vibrio, Photorhabdus, Erwinia*
CsrB	no significant hit	*Shigella, E. coli, Salmonella, Yersinia, Erwinia, Photorhabdus*
		Photobacterium, Pectobacterium, Vibrio, Aeromonas
DsrA	no significant hit	*Shigella, E. coli, Salmonella, Klebsiella*
RprA	no significant hit	*Shigella, E. coli, Salmonella, Photorhabdus, Erwinia, Yersinia*
