# Supplementary material for: Identification of chromosomal alpha-proteobacterial small RNAs by comparative genome analysis and detection in Sinorhizobium meliloti strain 1021
Source: BMC Genomics. 2007 Dec 19;8:467. doi: 10.1186/1471-2164-8-467 (PMC2245857; doi:10.1186/1471-2164-8-467)
Supplement: Additional file 3 — sRNAPredict outputs. The data provided presents the results obtained with sRNAPredict on S. meliloti's chromosome. [file 1471-2164-8-467-S3.pdf]

Number	Upstream ORF Name/Function	Dist. to Up_ORF	sRNA location	sRNA length	Dist. to Dn_ORF	Downstream ORF Name/Function	BLASTscore	BLASTexpect	Comment
**COMPARISON WITH AGROBACTERIUM**									
Pred1	SMc01933/Hypothetical	181	1398286-1398645	359	169	SMc01934/prolyl-tRNA synthetase	719	4.1e-25	***small RNA***
Pred2	SMc01431/acetolactate synthase 3	61	2276591-2276747	156	129	SMc01432/Hypothetical	585	9.5e-19	*ilvI* 5'UTR
Pred3	SMc02725/Anthranilate synthetase	53	2575840-2575986	146	73	SMc02726/Iron_transport	303	6.6e-06	*trpE* 5' UTR
Pred4	SMc02217/O-succinylhomoserine sulfhydrylase	28	580096-580261	165	**3**	SMc02218/2'-deoxycytidine 5'-triphosphate	577	3.8e-18	*metZ* 5' UTR
Pred5	SMc01283/50S ribosomal protein L17	28	1493333-1493770	437	**0**	SMc01282/Hypothetical	507	1.1e-22	*5'-3' UTR/ Sm-4*
Pred6	SMc03100/Hypothetical	49	3243325-3243519	194	20	SMc03099/Adenylate cyclase	434	6,00E-12	*repeat Sm-4*
Pred7	SMc01333/peptide chain release factor 2	88	1450536-1450928	392	18	SMc01332//Hypothetical	296	7.4e-06	*repeat Sm-1*
**COMPARISON WITH RHIZOBIUM ETLI**									
Pred8	SMc02050/trigger factor	37	1670130-1670375	245	3	SMc02049/glycine dehydrogenase	324	1.1e-06	*RIME*
Pred9	SMc02050/trigger factor	17	1670110-1670314	204	64	SMc02049/glycine dehydrogenase	324	1.1e-06	*RIME*
Pred10	SMc01722/GTP-binding protein	212	480343-480473	130	8	SMc01721/putative inner membrane	321	1.4e-06	*repeat Sm-5*
Pred1	SMc01933//Hypothetical	181	1398286-1398738	452	76	SMc01934/prolyl-tRNA synthetase	997	1.8e-37	***small RNA***
Pred2	SMc01431/acetolactate synthase 3	61	2276591-2276814	223	62	SMc01432/Hypothetical	468	2.8e-13	*ilvI* 5'UTR
Pred3	SMc02725/Anthranilate synthetase	53	2575840-2575986	146	73	SMc02726/Iron_transport	369	1.1e-08	*trpE* 5' UTR
Pred4	SMc02217/O-succinylhomoserine sulfhydrylase	28	580096-580263	167	**1**	SMc02218/2'-deoxycytidine 5'-triphosphate	650	3,00E-21	*metZ* 5' UTR
Pred5	SMc01283/50S ribosomal protein L17	28	1493333-1493769	436	**1**	SMc01282/Hypothetical	303	3.7e-09	*5'-3' UTR/ Sm-4*
Pred6	SMc03100/Hypothetical	49	3243325-3243473	148	66	SMc03099/Adenylate cyclase	307	6.9e-06	*repeat Sm-4*
Pred11	SMc02503/Hypothetical	26	3290285-3290536	251	78	SMc02502/ATP synthase	265	7.4e-08	*RIME*
Pred12	SMc02910/transport	103	267022-267105	83	35	SMc02911/Hypothetical	309	6.7e-06	*smc02911-5'UTR*
Pred13	SMc00018/ribonuclease H	33	1001412-1001580	168	8	SMc00072/peroxiredoxin	265	3.8e-08	*RIME*
Pred7	SMc01333/peptide chain release factor 2	88	1450536-1450945	409	**1**	SMc01332/Hypothetical	307	7,00E-16	*repeat Sm-1*
