# Supplementary material for: Identification of chromosomal alpha-proteobacterial small RNAs by comparative genome analysis and detection in Sinorhizobium meliloti strain 1021
Source: BMC Genomics. 2007 Dec 19;8:467. doi: 10.1186/1471-2164-8-467 (PMC2245857; doi:10.1186/1471-2164-8-467)
Supplement: Additional file 10 — Short (FASTA) alignment and corresponding synteny results. The data provided shows the results of the search for small regions of homology between alpha-proteobacteria. [file 1471-2164-8-467-S10.pdf]

<u>**Short Identity and synteny results**</u>

Syntenic genes are represented in yellow

➨ *sra07 and sra18*

```
               1       10        20        30        40        50
               |--------+---------+---------+---------+---------|
         sra07 GTCGATTTATTTGCCGTAATGTG---------------ACCCCTCCCTAA
         sra18      CGCCTTTGCCGAGGTGTGGCGAAGTACGTCCAGACCCCTCCCCAA
R.leguminosarum         TTGAGGAGATGAGCTGCAACCTGCGCCCAAACCCCTCCCCAA
        R.etli                                    TAGACCCCTCCCCAA
     Consensus .........tttgccga.gtgtg............cagACCCCTCCCcAA

               51      60        70        80        90        100
               |--------+---------+---------+---------+---------|
         sra07 CCCCTCCCCACAAGGGGGAGGGGCTACTTCTGCCGCACCTCGCGGGTCCA
         sra18 CCCCTCCCCACAAGGGGGAGGGGCGACTTCTGCAGCACTCTCTCGGCCAA
R.leguminosarum CCCCTCCCCACAAGGGGGAGGGGCTAA------CCCGTGGCGCCGCCTCG
        R.etli CCCCTCCCCACAAGGGGGAGGGGCTTAACGTGCGGCGCTCGCCGTATTCG
     Consensus CCCCTCCCCACAAGGGGGAGGGGCtaattctgccgCactccccggccca

               101     110       120       130       140       150
               |--------+---------+---------+---------+---------|
         sra07 AAGTCAGCCTTTGCGTCATCCGCGAACTTGCGAAATGGGAAAGCGCTAG-
         sra18 AGAACCAGCTTTGCGTCAACTTCGAGCTTGCAAAATGGCCGGGCGATCGG
R.leguminosarum CAAACTAGGCCTGCTGCATGCCCTGCGCTGGAAGATGAATGCGGCGAGGG
        R.etli CTCTCGGGGCCGCAGCATATTATGCATCCGGCTTGTTGTTTTGCGAGGCG
     Consensus aaaaC.agccctgcgtcaacc.cgaacctGcaaaaTgg.tg.Gcgatggg

               151     160       170       180       190       200
               |--------+---------+---------+---------+---------|
         sra07 CCGCAAGCGCCTAGCCCTTCCCCCTTGTGGGGAGGGGTTAGGGAGGGGCC
         sra18 CCCCGGGCGCCTAGCCCCTCCCACCTGTGGGGAGGGGTTGGGGAGGGGCC
R.leguminosarum CCGC--GCGTTAAGTCCCTCCCCCTTGTGGGGAGGGGTTGGGGAGGGGCC
        R.etli GTGCCACAGCGTAGCCCCTCCC-CTTGTGTGGAGGGGTTGGGGAGGGG
     Consensus ccgC.agcGcctAGcCCcTCCCcCtTGTGgGGAGGGGTTgGGGAGGGGcc

               201     210       220       230      238
               |--------+---------+---------+--------|
         sra07 TCATCCAACCGCCGTGGGCAGCGTGGCAATCACGCGAT
         sra18 TTATGCGACCGACCAGATCACGCGCG
R.leguminosarum TT
        R.etli
     Consensus tt....................................
```

	Left	Right
sra07	*smc00325*	*smc00326*
sra18	*smc03026*	*flgB*
R. etli	*RHE_CH01163*	*RHE_CH01164*
R. leguminosarum	*RL1293*	*RL1294*

➨ *sra08*

```
               1       10        20        30        40        50
               |--------+---------+---------+---------+---------|
         sra08 CTCAACCCCAGGCTTACCCCTCCCCAACCCCTCCCCACAAGGGGGAGGGG
        R.etli              ACCCCGCCCCAAACCCCTCCTCACAAGGGGGAGGGG
R.leguminosarum               ACCCCGCCCCAACCCCTCCCCACAAGGGGGAGGGG
     Consensus ...............aCCCc.CCCcAACCCCTCCcCACAAGGGGGAGGGG

               51      60        70        80        90        100
               |--------+---------+---------+---------+---------|
         sra08 CTTAACTTGCCTTACCGTCACCGCGTCGCCATCGTTTTGGCGCGGAGAGT
        R.etli CTTAACCTGCCGCACCGTTTCGTAAATATCTCGGCGTTTCGGGCGTAAGT
R.leguminosarum CTTAACTTGCCGCACCCGCTTTCCAGATTTTCGACGTTTCGGGAG-GAGC
     Consensus CTTAACtTGCCgcACCgtctc..ca...tctcggcgTTtcgGg.G.gAGt

               101     110       120       130       140       150
               |--------+---------+---------+---------+---------|
         sra08 GCCGCGGGGAAGCCCCTCCCCCTTGTGGCCCTTGTGGGGAGGGGTTGGGG
        R.etli GCGACGGTGCCTCTCCAAGCCCCTCC--CCCTTGTGGGGAGGAGTTGGGG
R.leguminosarum GGGTCGGGGCCTGAGCATATTCCCCTCCCCCTTGTGGGGAGGGGTG
     Consensus Gcg.CGGgGcctc.cCa..ccCctct..CCCTTGTGGGGAGGgGTtgggg

               151     160       169
               |--------+--------|
         sra08 AGGGGTTGCGGACGCAGAT
        R.etli AGGGGA
R.leguminosarum
     Consensus agggg..............
```

	Left	Right
sra08	*cfaI*	*smc00359*
R. etli	*kupch1*	*RHE_CH00862*
R. leguminosarum	*cheW*	*RL3290*

➨ *sra15*

```
               1       10        20        30        40        50
               |--------+---------+---------+---------+---------|
    R.meliloti            GGGTGCATCGGTCAATTGAAAATCCGCGTATCGGTGGTT
A.tumefaciens  GATAGCTCAGTTGGTAGAGCAGCGGATTGAAAATCCGCGTGTCGGTGGTT
        M.loti                         GACTGAAAATCCGCGTGTCGGTGGTT
     Consensus ............ggt..a.c.g..gAtTGAAAATCCGCGTgTCGGTGGTT

               51      60        70        80      88
               |--------+---------+---------+-------|
    R.meliloti CAAATCCGCTTATGGGCACCATTTTCTTTTTTGGGGGG
 A.tumefaciens CAAATCCGCCTCCGGGCACCATTTCTTTC
        M.loti CGAATCCGCCTCTGGGCACCATCCCCTCTTCTC
     Consensus CaAATCCGCcTctGGGCACCATttccTttt.t......
```

	Left	Right
sra15	*smc04432*	*tRNA-phe*
A.tumefaciens	*AGR_C_892*	*tRNA-phe*
M. loti	*mll6432*	*mll6433*

➨ *sra21*

```
           1       10        20        30        40        50
           |--------+---------+---------+---------+---------|
    sra21  ATCGCTCAGTCCCCAAACTCGCGCTCGATCTGCTGCGTAACTTATACGCG
     agro           TCCGCAATATTTCGCAC------------------------
Consensus  .........TCCcCAAaaTcgCGCaC.........................

           51      60        70        80        90        100
           |--------+---------+---------+---------+---------|
    sra21  TAAACGGTAAGATATACAATCACGGCATTGTGATCGCAGCTTTGGCGCAG
     agro  --------------------------------------------------
Consensus  ..................................................

           101     110       120       130       140       150
           |--------+---------+---------+---------+---------|
    sra21  GAACGCATGCAACGCCGCAATGTTCCGGAAGCCCGCACGCGCGCGGTCTC
     agro  -------------------------CGGAAATCCTGACCGGCGCGCTGCC
Consensus  .........................CGGAAacCCgcACccGCGCGcTccC

           151     160       170       180       190       200
           |--------+---------+---------+---------+---------|
    sra21  GCGGCTTGTCGCCGCCCTTGCCGCCCCTGCCATGATCCGGTAGGGAAGGC
     agro  GCCGCTTCTTTTCGCGCTTGCCGCCACCGCCATGAT-------GGATGTG
Consensus  GCcGCTTcTcgcCGCcCTTGCCGCCaCcGCCATGAT.......GGAaGgc

           201     210       220       23032
           |--------+---------+---------+-|
    sra21  ACCGACCAAGCTTGAAGTCCGGAATGCTTTCA
     agro  AACCTC
Consensus  AaCcaC..........................
```

	Left	Right
sra21	*smc00931*	*hisS*
A.tumefaciens	*AGR_C_3323*	

➨ *sra31*

```
                   1       10        20        30        40        50
                   |--------+---------+---------+---------+---------|
            sra31  TGCATGCCGCCCAAAAGTGTGCAGCGGTTTTGGGATAACGACATGCATGA
R.leguminosarum    TGCTTGTCGCCCAAAAGTGTGCAGCGGTTTTGGGACAACGACATGCATCA
           M.loti  TGCATGTCGCCCAAAAGTGTGCAGCGGTTTTGGGAGAACGACATGGATCA
           R.etli  TGCATGTAGCCCAAAAGTGTGCAGCGGTTTTGGGGGCACGACATGCATAA
        Consensus  TGCaTGtcGCCCAAAAGTGTGCAGCGGTTTTGGGagaACGACATGcATcA

                   51      60        70        80        91
                   |--------+---------+---------+--------+|
            sra31  T---AACGACCTACAGCGCGGCGCGCTTTATGTCCTAGGCC
R.leguminosarum    AG-CAAGGACCTGAAGCGCGTCGCGCTTTAGA
           M.loti  AAACAAAGAGCTAAAGCGCATCGCC
           R.etli  A---AAGACCCTCAAGCGCCTCGCA
        Consensus  a...AAggacCTaaAGCGCgtCGCg................
```

	Left	Right
sra31	*smc01806*	*smc01807*
R. etli	*RHE_CH02389*	*RHE_CH02390*
R. leguminosarum	*RL0960*	*RL0961*
M. loti	*mll0880*	mlr0883

➨ *sra34*

```
                 1       10        20        30        40        50
                 |--------+---------+---------+---------+---------|
          sra34  TTCCGGTTCGGATCGGACTTCGAACCCCGCGGGCAACCGCGGGGTTTTTC
           mezo                GAAATCGGAACCCGGCGGGCAACCGCCGGGTTTTTT
           etli           TCCACAATCAGGAAGACCCCGCTGGAAACAGCGGGTTTTTC
  leguminosarum            CCGCAATTATGAAGACCCCGCTGGAAACAGCGGGTTTTTC
      Consensus  ..........ccacaataatgaaaACCCcGCgGGaAACaGCgGGGTTTTTc

                 51      60        70        80      88
                 |--------+---------+---------+-------|
          sra34  TTTTGGCGATTACCAGAAC
           mezo  CATTTGCTAGTACAAGCTGCACTACTATGCGAGCGAGT
           etli  GTTTTTGATG
  leguminosarum  ATTTTCATGA
      Consensus  .tTTtgctag............................
```

	Left	Right
sra34	*tufA*	*smc01325*
R. etli	*tufB*	*RHE_CH01659*
R. leguminosarum	*tufB2*	*RL1758*
M. loti	*mlr4028*	*mll4029*

➨ *sra36*

```
                 1       10        20        30        40        50
                 |--------+---------+---------+---------+---------|
     R.meliloti             GGGTGCATCGGTCAATTGAAAATCCGCGTATCGGTGGTT
A.tumefaciens    GATAGCTCAGTTGGTAGAGCAGCGGATTGAAAATCCGCGTGTCGGTGGTT
         M.loti                          GACTGAAAATCCGCGTGTCGGTGGTT
      Consensus  ............ggt..a.c.g..gAtTGAAAATCCGCGTgTCGGTGGTT

                 51      60        70        80      88
                 |--------+---------+---------+-------|
     R.meliloti  CAAATCCGCTTATGGGCACCATTTTCTTTTTTGGGGGG
A.tumefaciens    CAAATCCGCCTCCGGGCACCATTTCTTTC
         M.loti  CGAATCCGCCTCTGGGCACCATCCCCTCTTCTC
      Consensus  CaAATCCGCcTctGGGCACCATttccTttt.t......
```

	Left	Right
sra15	*smc04432*	*tRNA-phe*
A.tumefaciens	*AGR_C_892*	*tRNA-phe*
M. loti	*mll6432*	*mll6433*

➨ *sra37*

```
                   1       10        20        30        40        50
                   |--------+---------+---------+---------+---------|
            sra37   GGTAACCACGCTTATAAGCGGTTGTGAATGTGACGATTCAGCAACACAT
R.leguminosarum    CGGTCGCAAGACAAATCGACGTTCCAATTTGTGACGATTCAGCAACACAT
           R.etli                              TTGTGACGATTCAGCAACAGTT
        Consensus  .ggt..c.a..c..at...cg.t.....tTGTGACGATTCAGCAACAcaT

                   51      60        70        80        90        100
                   |--------+---------+---------+---------+---------|
            sra37  TTCCTGCTACGGTTTGCTACTATCCGGACCGGGTCGATTATGTCCATTTC
R.leguminosarum    TTT-TACGACGTTTGGCGTATGGTGGCGCCAGGGCGAATTTGTCCATTTC
           R.etli  TTT-TACGATGAATGGCGTATGGTGGCGATGGGGCGAATTTGTCCATTTC
        Consensus  TTt.TaCgAcG.tTgGCgtaTggtgGcgccgGGgCGAaTtTGTCCATTTC

                   101     110       120   126
                   |--------+---------+-----|
            sra37  CCGCCACAAAATGAGAGCAGCGATA
R.leguminosarum    CCGCCACAAAGAGGGCGCAGCGCTAT
           R.etli  CCGCCACAA
        Consensus  CCGCCACAAa..g.g.gcagcg.ta.
```

	Left	Right
sra37	*smc02079*	*exoR*
R. etli	*RHE_CH01813*	
R. leguminosarum	*RL2036*	*exoR*

➡ sra42

```
             1       10        20        30        40        50
             |--------+---------+---------+---------+---------|
       sra42 GGGAAAATGGCGGACGCATGGAAAAGGGCCCCGTGATGCTTGCGGCAGTC
      R.etli         GTTGACCGCAGCATAATAGAGTCCTGTCTGCCGGCGGCATTG
      M.loti                                      CCTTGCGGCAGTC
   Consensus ........g..g..cgca....aa..g...cc....tgCttGCGGCAgTc

             51      60        70        80        90        100
             |--------+---------+---------+---------+---------|
       sra42 GAGACAATGGCAGAGGCCTACCCGGTATGGTCGGCCCGTCGCCACAAATC
      R.etli GGAACAGTGGCAACCGACGACAGGCCCGGGCTTTGCCCGAATTGAAAATC
      M.loti CTTGCAGTGACAAAGGCCGACGCGTGTCGGCTGGCCGGTGACGACGAAAT
   Consensus g..aCAgTGgCAaagGcCgAC.cG....GGctggcCcgt.ac.acaAAtc

             101     110       120       130 134
             |--------+---------+---------+---|
       sra42 GCACGTTGCCGCACAGGCAACCGCCTGGGAATCG
      R.etli GCACGGCGCCGCAAAGACAGCCGCCTGTAT
      M.loti GCACACCGCCGCACAGGCAACTGCCGGT
   Consensus GCACg.cGCCGCAcAGgCAaCcGCCtGt......
```

	Left	Right
sra42	*SMc01197*	*SMc01196*
R. etli	*RHE_CH02373*	
M. loti	*mlr1783*	

➡ sra44

```
                 1       10        20        30        40        50
                 |--------+---------+---------+---------+---------|
           sra44 GTCGTTCTCCAATCTCGCCGGATCCAAGGTCCGGCAGCTTGTCAATACGA
R.leguminosarum   TCATTCTCCAATCTCGCCGGATACAAGGTCCGGCAGCTTGTAGCGTTTG
       Consensus .TCaTTCTCCAATCTCGCCGGATaCAAGGTCCGGCAGCTTGTaaagacga

                 51      60        70        80        90        100
                 |--------+---------+---------+---------+---------|
           sra44 TGCAAGTATTCGCGCACCGCTTATGTAAT-GGCTAAAAGGCGTTTTTCAC
R.leguminosarum  GGCAATTGT---CTTGCCCTTTATGTAAGAGGCCAAAAGGGACTTTTCAG
       Consensus gGCAAgTaT...CgcaCCccTTATGTAAg.GGCcAAAAGGcacTTTTCAc

                 101     110       120       130       140       150
                 |--------+---------+---------+---------+---------|
           sra44 CCCATTTGCCAAGCGGAAAATTGCTTTTCCGCAATTTCATTCAATGCTCT
R.leguminosarum  CCCAATTGGCAAGCGGAAATTTGCATTTCCGCAAGTTTTGA
       Consensus CCCAaTTGcCAAGCGGAAAaTTGCaTTTCCGCAAgTTcaga.........

                 15155
                 |---|
           sra44 CCGCT
R.leguminosarum
       Consensus .....
```

	Left	Right
sra44	*smc00530*	*nifS*
R. etli	*sufB*	*nifSCh*
R. leguminosarum	*sufB*	*nifS*
A.tumefaciens	*AGR_C_3348p*	*nifS*

➡ sra67

```
                 1       10        20        30        40        50
                 |--------+---------+---------+---------+---------|
           sra67  GCCGGTAGCGCTCTAAAACAGCCGGCGTCTCATCGACCCGGCTGACGCA
          R.etli  GCCGGTAGCGCTCAAAAACAGCCGGCGTCTCATCGACCCGGCTG-GCCG
 R.leguminosarum  GCCGGTAGCGCTCAAAAACAGCCGGCGTCTCATCGACCCGGCTG-GCCG
   A.tumefaciens AGCCGGTAAGCGTCATATGCAGCCGGTGTCCCATCGACCCGGCTGAGACG
       Consensus .GCCGGTAgcgcTCaaAaaCAGCCGGcGTCtCATCGACCCGGCTG.gcCg

                 51      60        70        80        90        100
                 |--------+---------+---------+---------+---------|
           sra67 CCGCAAGGTTCAAAAGCCAGACTTCAAAGGGGAGATGGGGCGATACCCAA
          R.etli TACCAGGTTCAGTCCAGAAAAC-----AGGGGAGATGGGGCGCCATCTCC
 R.leguminosarum TATCAGGTTCAGTCTAGAAAAC-----AGGGGAGATGGGGCGCCATCTCC
   A.tumefaciens TATCAGGTTCAGTCAAGAAAAC-----AGGGGAGATGGGGCACCACCCAA
       Consensus tatCAgGtTcagtcaagaAaAC.....AGGGGAGATGGGGCgccAcCcaa

                 101     110       120       131
                 |--------+---------+---------+|
           sra67 CTCCCCTTTTCGCATGTCTGGAGGAAGAACC
          R.etli CCTTTTTCTTTGTCCAAAC
 R.leguminosarum CCTTTTT
   A.tumefaciens CTCCCCTTTTT
       Consensus CcccccTtttt....................
```

	Left	Right
sra67	*rpmA*	*smc03773*
R. etli	*rpmA*	*RHE_CH04064*
R. leguminosarum	*rpmA*	*rl4678*
A.tumefaciens	*rpmA*	*Atu2783*
