# Supplementary material for: Identification of chromosomal alpha-proteobacterial small RNAs by comparative genome analysis and detection in Sinorhizobium meliloti strain 1021
Source: BMC Genomics. 2007 Dec 19;8:467. doi: 10.1186/1471-2164-8-467 (PMC2245857; doi:10.1186/1471-2164-8-467)
Supplement: Additional file 11 — Artemis Comparison Tool (ACT) screenshots alignment and corresponding synteny results. The data provided presents the results of the ACT comparisons. [file 1471-2164-8-467-S11.pdf]

Exemple of Artemis Comparison Tool (ACT) results

Syntenic genes are in yellow

➡ *ffs= 4.5S*

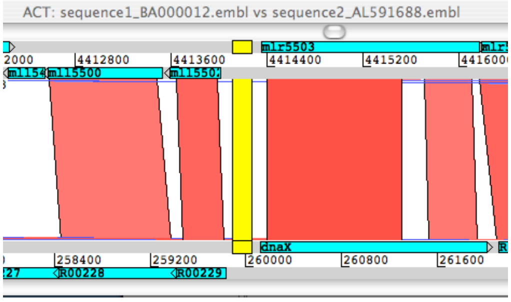

	Left	Right
sra05=ffs=4.5S	*SMc02904*	*dnaX*
R. etli	*RHE_CH00127*	*dnaX*
R. leguminosarum	*rl0135*	*dnaX*
A.tumefaciens	*AGR_C_148*	*dnaX*
M. loti	*mll5502*	*dnaX*

➡ *tmRNA*

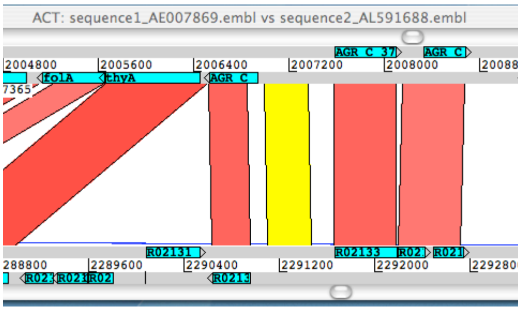

	Left	Right
sra49 = tmRNA	*SMc01449*	*SMc01450*
A.tumefaciens	*AGR_C_3711*	*AGR_C_3712*
M. loti	*mll1468*	*mll1469*

➡ *rnpB*

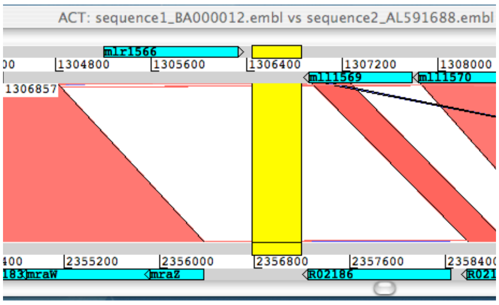

	Left	Right
sra50=tmRNA	*mraZ*	*SMc01856*
R. etli	*mraZ*	*RHE_CH02857*
R. leguminosarum	*mraZ*	*RL3317*
A.tumefaciens	*mraZ*	*AGR_C_3823*
M. loti	*mlr1566*	*mll1569*

➡ *sra02*

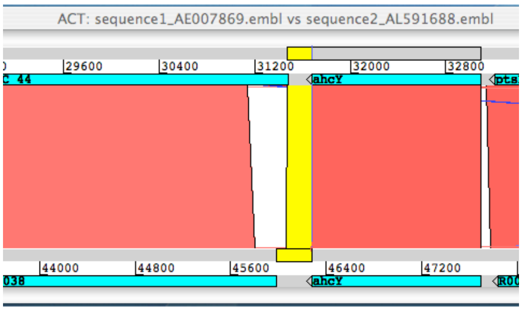

	Left	Right
sra02	*SMc02756*	*ahcY*
R. etli	*RHE_CH00030*	*ahcY*
R. leguminosarum	*RL0030*	*ahcY*
A.tumefaciens	*AGR_C_44*	*ahcY*
M. loti	*mll5087*	*ahcY*

➡ sra03

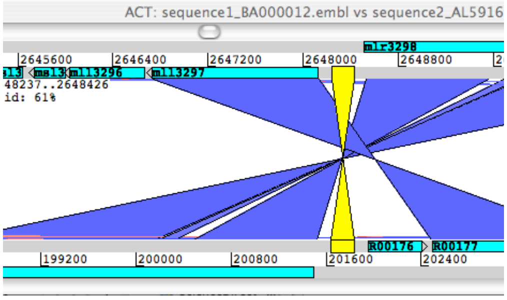

	Left	Right
sra03	*polA*	*SMc02851*
R. etli	*polA*	*RHE_CH00150*
R. leguminosarum	*polA*	*R00176*
A.tumefaciens	*AGR_C_168*	*AGR_C_167*
M. loti	*polA*	*mll3297*

➡ sra04

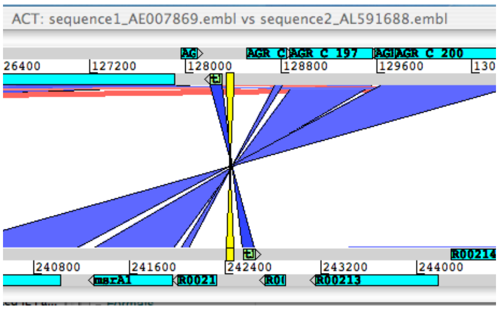

	Left	Right
sra04	*smc02886*	*tRNA_Ser*
A.tumefaciens	*AGR_C_196*	*tRNA_Ser*

➡ sra06

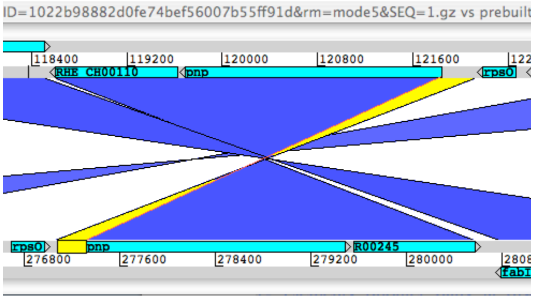

	Left	Right
sra06	*rpsO*	*pnp*
R. etli	*rpsO*	*pnp*
R. leguminosarum	*rpsO*	*pnp*
A.tumefaciens	*rpsO*	*pnp*
M. loti	*rpsO*	*pnp*

➡ sra09

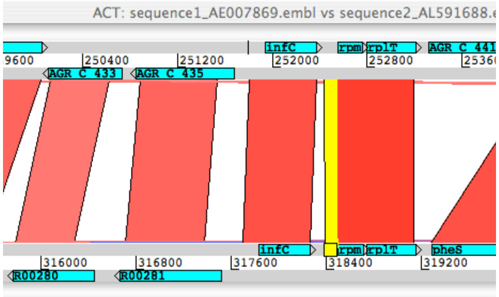

	Left	Right
sra09	*infC*	*rpmE*
R. etli	*infC*	*rpmE*
R. leguminosarum	*infC*	*rpmE*
A.tumefaciens	*infC*	*rpmE*

➡ sra13

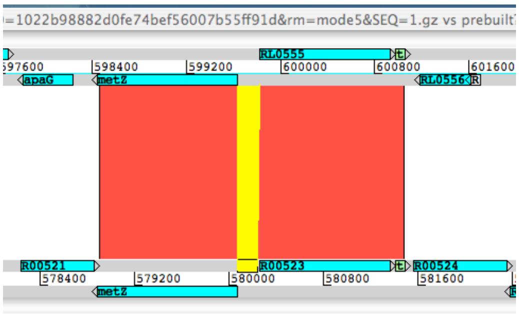

	Left	Right
sra13	*metZ*	*SMc02218*
R. etli	*metZ*	*dcd*
R. leguminosarum	*metZ*	*mlr5652*
A.tumefaciens	*metZ*	*dcd*
M. loti	*metZ*	*pnp*

➨ *sra16*

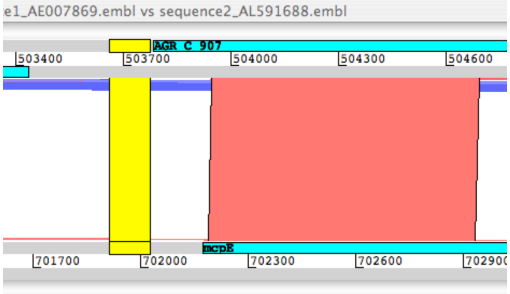

	Left	Right
sra16	*smc03003*	*mcpE*
A.tumefaciens	*AGR_C_905*	*AGR_C_907*

➨ *sra19*

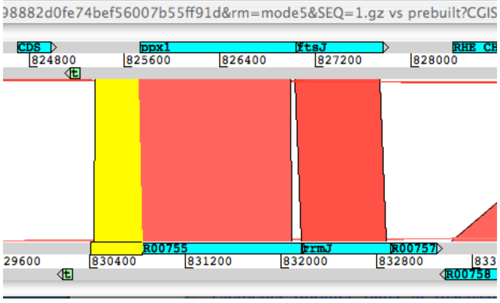

	Left	Right
sra19	*tRNA_Gln*	*SMc00810*
R. etli	*tRNA_Gln*	*ppx1*
A.tumefaciens	*AGR_C_1096*	*AGR_C_1098*

➨ *sra20*

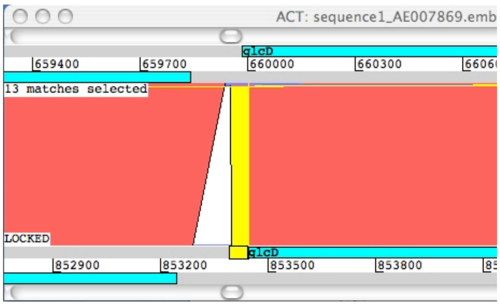

	Left	Right
sra20	*SMc00831*	*glcD*
R. etli	*RHE_CH00807*	*glcD*
A.tumefaciens	*AGR_C_1186*	*glcD*
R. leguminosarum	*RL0863*	*glcD*

➨ *sra22*

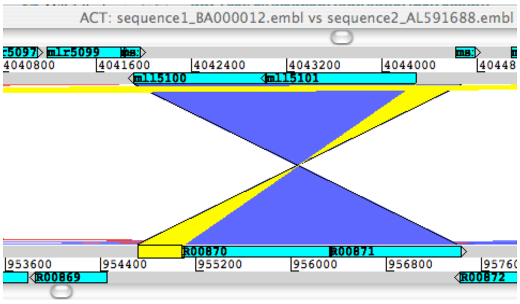

	Left	Right
sra22	*SMc00981*	*SMc00982*
M. loti	*msr5103*	*mll5101*

➨ *sra23*

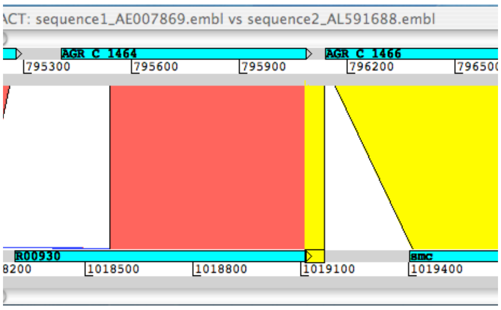

	Left	Right
sra23	*smc00023*	*smc*
A.tumefaciens	*AGR_C_1464*	*AGR_C_1466*

➡ sra25

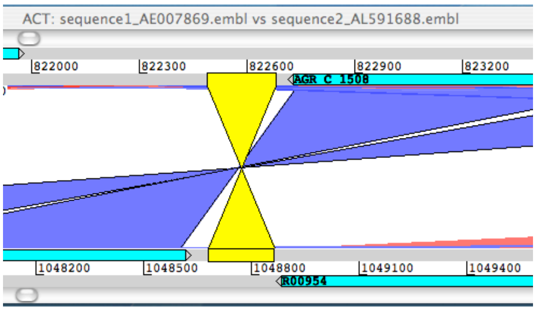

	Left	Right
sra25	smc00034	smc00096
A.tumefaciens	AGR_C_1508	cysA1

➡ sra26

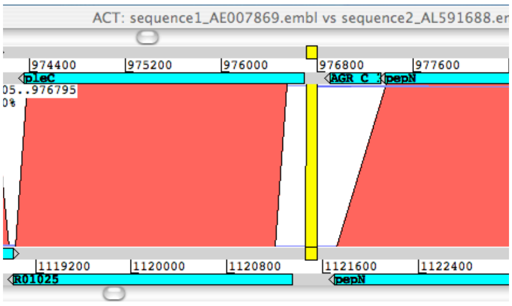

	Left	Right
sra26	smc02369	pepN
R. etli	RHE_CH01297	pepN
R. leguminosarum	RL1445	pepN
A.tumefaciens	pleC	AGR_C_892

➡ sra27

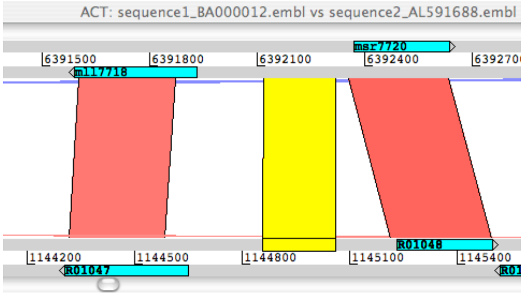

	Left	Right
sra27	smc02391	smc02392
A.tumefaciens	AGR_C_1859	AGR_C_1864
M. loti	mll7718	msr7720

➡ sra29

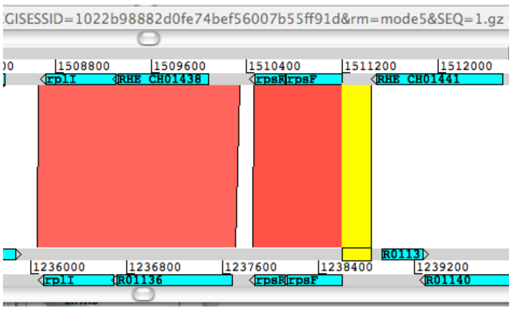

	Left	Right
sra29	rpsF	smc00569
R. etli	rpsF	RHE_CH01441
R. leguminosarum	rpsF	RL1556
A.tumefaciens	rpsF	AGR_C_2023
M. loti	rpsF	mlr7847

➡ sra32

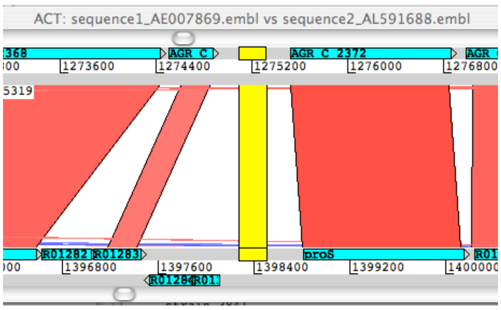

	Left	Right
sra32	SMc01933	proS
R. etli	RHE_CH01622	proS
R. leguminosarum	RL1718	proS
A.tumefaciens	AGR_C_2369	proS
M. loti	rpsF	proS

➡ *sra33*

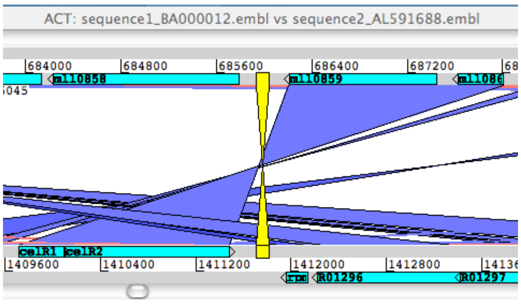

	Left	Right
sra33	*celR2*	*rpmG*
R. etli	*celR2*	*rpmG*
R. leguminosarum	*RL1730*	*rpmG*
A.tumefaciens	*celR2*	*AGR_C_2230*
M. loti	*celR2*	*mll0858*

➡ *sra35*

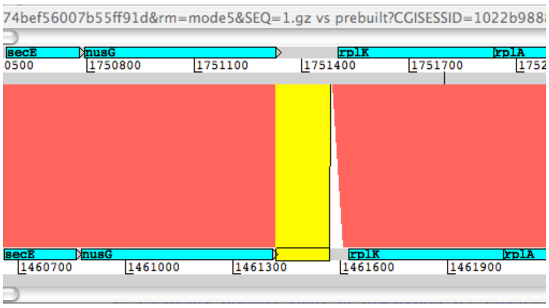

	Left	Right
sra35	*nusG*	*rplK*
R. etli	*nusG*	*rplK*
R. leguminosarum	*nusG*	*rplK*
A.tumefaciens	*nusG*	*rplK*
M. loti	*nusG*	*rplK*

➡ *sra36*

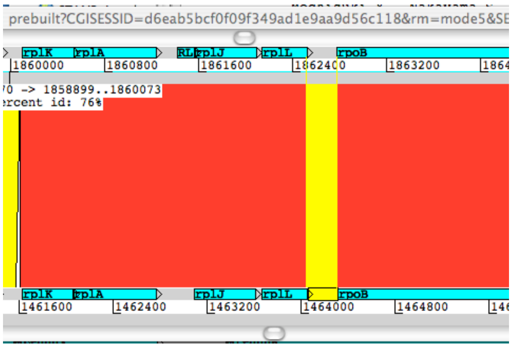

	Left	Right
sra36	*rplL*	*rpoB*
R. etli	*rplL*	*rpoB*
R. leguminosarum	*rplL*	*rpoB*
A.tumefaciens	*rplL*	*rpoB*

➡ *sra39*

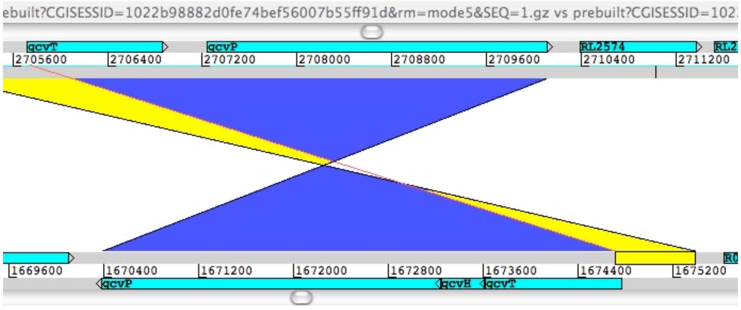

	Left	Right
sra39	*SMc01242*	*gcvT*
R. etli	*RHE_CH02240*	*gcvT*
R. leguminosarum	*RL2571*	*gcvT*
A.tumefaciens	*AGR_C_2705*	*gcvT*
M. loti	*mll0880*	*gcvT*

➡ *sra41*

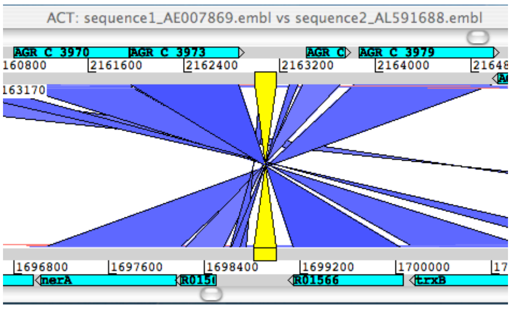

➨ sra45

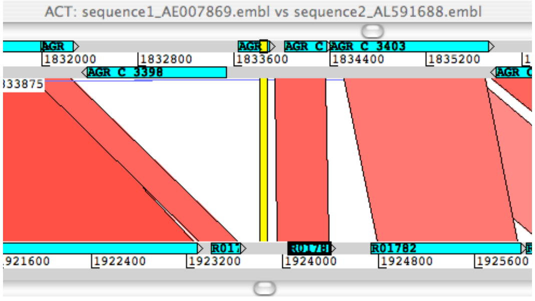

	Left	Right
sra45	SMc00487	SMc00538
R. etli	RHE_CH02303	
R. leguminosarum	RL2613	RL2614
A.tumefaciens	AGR_C_3399	

➨ sra46

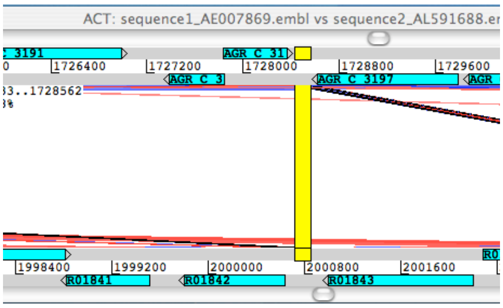

	Left	Right
sra46	SMc00165	SMc00164
R. etli	RHE_CH02368	aroG
R. leguminosarum	RL2681	RL2682
A.tumefaciens	AGR_C_3195	AGR_C_3197

➨ sra51

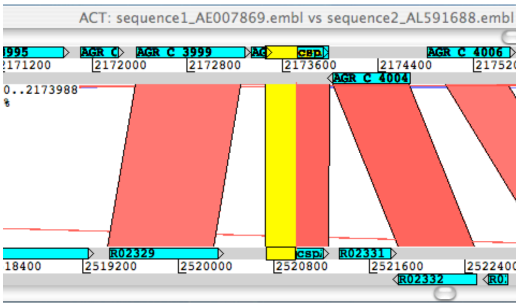

	Left	Right
sra51	SMc01584	cspA3
R. etli	RHE_CH03004	cspA5
R. leguminosarum	RL3448	cspA
A.tumefaciens	AGR_C_4001p	cspA

➨ sra54

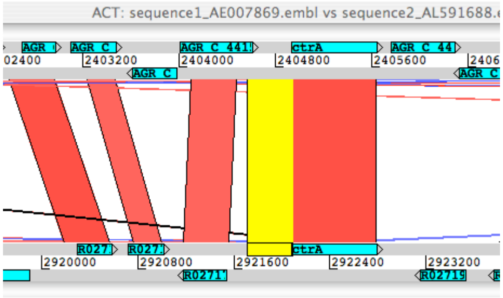

	Left	Right
sra54	SMc00655	ctrA
R. etli	RHE_CH03333	ctrA
A.tumefaciens	AGR_C_4415	ctrA

➨ sra55

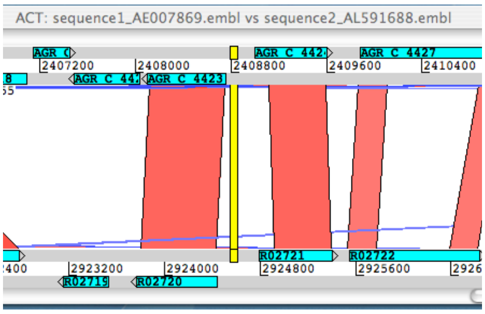

	Left	Right
sra55	SMc00652	SMc00651
A.tumefaciens	AGR_C_4423	AGR_C_4424

➡ *sra56*

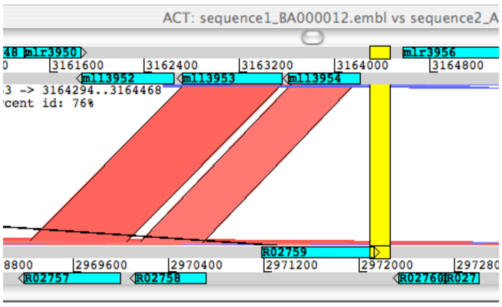

	Left	Right
sra56	*smc00530*	*nifS*
R. etli	*RHE_CH03492*	*RHE_CH03493*
R. leguminosarum	*RL4003*	*RL4004*
A.tumefaciens	*Linear Chom. Atu3734*	
M. loti	*mlr4028*	*mll4029*

➡ *sra58*

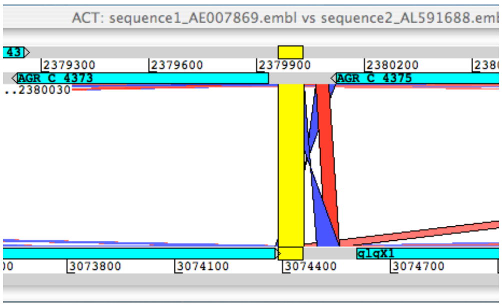

	Left	Right
sra51	*pgm*	*glgX1*
R. etli	*RHE_CH01194*	*RHE_CH01195*
R. leguminosarum	*RL3112A*	
