# Supplementary material for: Identification of chromosomal alpha-proteobacterial small RNAs by comparative genome analysis and detection in Sinorhizobium meliloti strain 1021
Source: BMC Genomics. 2007 Dec 19;8:467. doi: 10.1186/1471-2164-8-467 (PMC2245857; doi:10.1186/1471-2164-8-467)
Supplement: Additional file 5 — Normalized expression levels (log2SNR) of IGRs candidates. The data provided compiles the normalized expression levels for the candidates. [file 1471-2164-8-467-S5.pdf]

ID	Latence	Early-Exponential	Mid-Exponential	Late-Exponential	Statio
5S	2.4	10.76	2.80	2.87	3.85
sra05(*4.5S*)	12.4	16.14	7.02	2.25	26.31
sra49(*tmRNA*)	6.9	14.71	19.23	1.99	12.55
sra50(*rnpB*)	24.3	28.70	57.29	52.77	52.02
sra01(oriC)	1.4	1.47	1.32	1.16	1.09
sra02	1.2	1.09	1.45	1.09	1.24
sra03	4.2	1.85	4.09	5.38	3.26
sra04	3.0	2.53	3.16	1.57	1.78
sra06	1.6	1.08	1.28	1.10	0.94
sra07	1.2	1.16	1.09	0.89	1.05
sra08	1.1	1.13	1.03	1.11	0.88
sra09	1.4	0.96	1.39	1.18	1.29
sra10A	5.5	2.61	4.83	2.65	1.97
sra10B	3.8	1.44	4.33	2.11	2.07
sra11	1.5	1.40	1.49	1.48	1.20
sra12-A	1.5	1.02	1.49	1.22	1.50
sra12-B	5.0	4.33	3.33	3.21	4.56
sra13	0.9	1.02	1.01	0.80	1.00
sra14-A	9.0	2.84	8.67	4.14	3.73
sra14-B	12.5	6.32	10.62	5.89	3.73
sra15	4.9	2.32	3.46	6.33	3.45
sra16	1.9	1.39	2.50	3.16	1.83
sra17	2.6	1.98	2.55	1.88	1.69
sra18	1.8	1.26	2.19	1.41	1.13
sra19	1.0	1.26	1.12	0.92	1.58
sra20	1.3	1.28	1.17	1.10	0.93
sra21	1.6	1.47	1.84	1.28	1.11
sra22-A	1.2	0.92	1.16	1.35	0.95
sra22-B	1.9	1.01	1.09	1.00	1.15
sra23	1.1	1.27	1.01	1.03	0.88
sra24	1.2	1.11	1.38	1.32	0.96
sra25	1.5	1.32	2.09	1.45	1.57
sra26	2.0	1.24	1.84	1.91	1.76
sra27	1.1	1.08	1.17	1.08	0.89
sra28-A	5.8	2.86	7.39	3.72	3.04
sra28-B	3.9	2.32	3.50	2.94	2.06
sra29	3.6	1.10	2.22	1.24	1.65
sra30	1.6	1.21	1.89	1.44	1.25
sra31	1.1	1.11	1.13	1.22	1.12
sra32-A	3.5	5.25	12.02	1.86	2.27
sra32-B	1.6	1.16	1.63	1.02	1.09
sra33	2.3	16.16	2.29	2.50	2.49
sra34	1.3	1.18	1.28	1.21	1.42
sra35	15.0	2.91	14.41	2.08	3.41
sra36	2.4	3.43	2.17	1.43	2.17
sra37	1.7	1.28	1.70	1.15	1.08
sra38A	1.4	1.08	1.53	1.25	1.47

sra38D	1.8	1.17	1.53	1.44	2.03
sra39A	5.1	19.35	20.80	5.09	6.03
sra39B	3.4	3.40	2.05	1.72	4.82
sra40A	1.6	1.00	1.53	1.63	1.23
sra40B	1.5	1.43	1.90	1.66	1.21
sra41	7.5	3.89	9.13	6.98	7.22
sra42	2.1	1.25	2.17	1.57	1.25
sra43-A	3.1	1.67	2.52	3.23	2.19
sra43-B	3.8	2.21	2.52	2.04	3.11
sra44	1.2	1.23	1.28	1.16	1.31
sra45	3.7	3.72	3.13	2.03	1.87
sra46	1.5	1.11	1.61	1.14	0.97
sra47-A	6.3	3.16	7.35	3.30	3.66
sra47-B	10.8	12.50	16.01	4.45	5.82
sra48	4.3	1.85	3.79	1.85	2.48
sra49	6.9	14.71	19.23	1.99	12.55
sra50A	4.4	2.67	5.65	3.78	3.70
sra50B	24.3	28.70	57.29	52.77	52.02
sra51A	3.6	2.19	6.56	5.88	3.14
sra51B	3.1	2.09	2.57	1.96	1.52
sra52	2.1	1.33	2.17	1.57	1.26
sra53A	3.1	2.78	3.44	2.59	1.95
sra53B	3.2	2.05	2.85	2.80	1.54
sra54	2.1	1.69	2.20	1.65	1.26
sra55	6.1	3.06	5.65	3.78	2.26
sra56	3.1	2.36	2.98	1.68	2.63
sra57	1.6	1.43	1.36	1.46	1.21
sra58	2.4	1.37	1.81	1.62	1.49
sra59	18.9	9.83	13.57	5.24	10.58
sra60A	3.4	2.92	4.15	6.98	3.75
sra60B	5.0	2.95	4.76	2.89	2.38
sra61	1.8	1.17	1.58	1.24	1.22
sra62A	26.0	24.86	49.22	18.10	27.05
sra62B	5.6	5.87	13.71	10.70	11.51
sra63A	1.6	1.82	1.68	1.96	1.57
sra63B	1.4	1.04	1.71	1.66	2.62
sra63C	2.5	1.74	1.61	1.51	1.82
sra63D	1.2	1.17	1.15	1.28	1.40
sra64	1.1	1.10	1.27	1.35	1.22
sra65A	4.3	1.09	1.37	1.21	1.16
sra65B	1.7	1.14	1.60	1.39	1.22
sra66	1.2	1.22	1.88	1.45	1.40
sra67	3.8	1.48	3.32	1.59	1.43
