# Supplementary material for: Identification of chromosomal alpha-proteobacterial small RNAs by comparative genome analysis and detection in Sinorhizobium meliloti strain 1021
Source: BMC Genomics. 2007 Dec 19;8:467. doi: 10.1186/1471-2164-8-467 (PMC2245857; doi:10.1186/1471-2164-8-467)
Supplement: Additional file 12 — TargetRNA results. The data provided shows the results of the target analysis for each valid sra gene. [file 1471-2164-8-467-S12.pdf]

Proposed alignment from TargetRNA analysis of *sra* genes interactions ($e < 10^{-3}$)

➨ *sra32 targets*

1- fliM : Flagellar motor switch transmembrane protein

Score: -84 Pvalue: 0.000199501

```
sRNA            60   AUCUUAGGCGCGC-UUG--UUCGCUGCCGCCG    88
                     || |:|||| ||| |||  |||||||||||||
mRNA (fliM)     2    UACAGUCCG-GCGGAACGAAAGCGACGGCGGC   -29
```

2- **SMc01800 =** transmembrane cytochrome C oxidase

Score: -83 Pvalue: 0.000237386

```
sRNA            69   GCGCUUGUUCG-CUGCCGCCGGAAACGG    95
                     ||:||| :||: ||| ||||||||||||
mRNA (SMc01800) -2   CGUGAAGGAGUAGACAGCGGCCUUUGCC   -29
```

➨ *sra33 targets*

1- SMc00899 = hypothetical protein

Score: -78 Pvalue: 0.000301856

```
sRNA            2    CCAUGAUGCUCAGGUCCGCCGCAU-CU-CCUGGGUCGUGGGGUCGGUC    47
                     || ||||||||||  ||: |: || || ||||| || ||   |||:||
mRNA (SMc00899) 19   GGAACUACGAGUCGCGGUAGUCUACGAAGGACCAAGGAC---AGCUAG   -26
```

2- **rkpJ =** capsular polysaccharide biosynthesis/export protein

Score: -73 Pvalue: 0.000749566

```
sRNA            40   GGUCG-GUCGGCUGGCCUCGGACUUGCGGAUUCC    72
                     ||||| :|| |||| | ||||    |||||||||
mRNA (rkpJ)     18   CCAGCAUAGACGACAGUAGCC----CGCCUAAGG   -12
```

➨ *sra41 copie 1 target*

1- SMc02392 = hypothetical protein

Score: -75 Pvalue: 0.00097857

```
sRNA            84   AAGGGC-CCAAGUUUUUCUUGUGGCCCU--CUUUUUUU    118
                     |||:|  |||  ||||||||||  ||||  |||||:||
mRNA (SMc02392) 11   UUCUCACGGU--AAAAAGAACA--GGGACAGAAAAGAA    -23
```

➨ *sra41 copie 2 target*

1- SMc00317 = hypothetical protein

1 SMc00317 hypothetical protein

Score: -80 Pvalue: 0.000214469

```
sRNA            23   CUCCCCAGCCGCUGCAGCAGCUGUUCC    49
                     |||||  |||||||||| |||  ||||
mRNA (SMc00317) -4   GAGGGAGCGGCGACGUCUUCGCGAAGG   -30
```

1- SMc01118 = hypothetical protein

2 SMc01118 hypothetical protein

Score: -76 Pvalue: 0.000443528

```
sRNA            5    GCAUCU-AGCGGCUUUCUCCUCCCCAGCCGCUGC    37
                     |||| | ||| ||  |||||||||| ||  ||||
mRNA (SMc01118) 4    CGUAAAGUCGGCGCCAGAGGAGGGGACGCGGACG   -30
```

➡ *sra12b targets*

1- SMc03123 = hypothetical transcriptional regulatory protein

```
Score: -91              Pvalue: 0.000110731

sRNA               50   AUCAGAAAUUCUGCUGCCCC-U-CAUCCUC--UGCG-CGCUGUUCGAUUGU   95
                        |||||   |||||||||||| | ||:||:|  |||: |||||: || :|||
mRNA (SMc03123)    20   UAGUCA--AAGACGACGGGGUACGUGGGGGGGACGUUGCGACGCGCAGACA   -29
```

2- purU2 = formyltetrahydrofolate deformylase

```
Score: -79              Pvalue: 0.000821004

sRNA               54   GAAAUUCUGC-UGCCCCUCAUC-CUCUGCGC--GCUGUUCGAUUGUACGGG   100
                        |||||||||| ||    ||||  |||| | |  ||:|||||  :|:|||||
mRNA (purU2)       18   CUUUAAGACGUACA---AGUAAAGAGAAGGGAGCGGCAAGCGCGCGUGCCC   -30
```

➡ *sra34 targets*

1- SMc00409 = hypothetical signal peptide protein

```
Score: -96              Pvalue: 7.56916e-05

sRNA               87   UGGGGCG--AUCUCUUUCGCGUGUGAUGUCU   115
                        ||||:||  || :|||||| |||||||||||
mRNA (SMc00409)    1    ACCCUGCAAUACGGAAAGCUCACACUACAGA   -30
```

2- SMc03128 = transport system permease ABC transporter protein

```
Score: -93              Pvalue: 0.000123148

sRNA               63   CCAGAUCAACGCUUUUUGAAGCGGUGGGGCGAUCUCUUUCGCGUGUGAUGUCUG   116
                        |||||||||||      ||||| :|:| || |||||:|| | :| |:|||:|:|
mRNA (SMc03128)    19   GGUCUAGUUGC------CUUCGGUAUCGCG-UAGAGGAACC-UAGAUUACGGGC   -27
```

➡ *sra66 targets*

1- tolR = putative transport transmembrane protein

```
Score: -84              Pvalue: 0.000756567

sRNA               172  GCUAAAUGCACAUACCCCUCCAUGCUUGG---CCGUGCUUUCUGGGCG   216
                        ||:|| ||| ||||||| || | || |||   |||||||||  || ||
mRNA (tolR)        18   CGGUUGACG-GUAUGGGUAG-U-CGCACCAGAGGCACGAAACUCCAGC   -27
```
