# Supplementary material for: Identification of chromosomal alpha-proteobacterial small RNAs by comparative genome analysis and detection in Sinorhizobium meliloti strain 1021
Source: BMC Genomics. 2007 Dec 19;8:467. doi: 10.1186/1471-2164-8-467 (PMC2245857; doi:10.1186/1471-2164-8-467)
Supplement: Additional file 13 — Alifold and RNAz secondary predictions. The data provided presents the alternative structures predicted for sra genes depending on the tools used. [file 1471-2164-8-467-S13.pdf]

## Predicted structure of alpha-proteobacteria conserved *sra* genes

*sra05 = ffs*

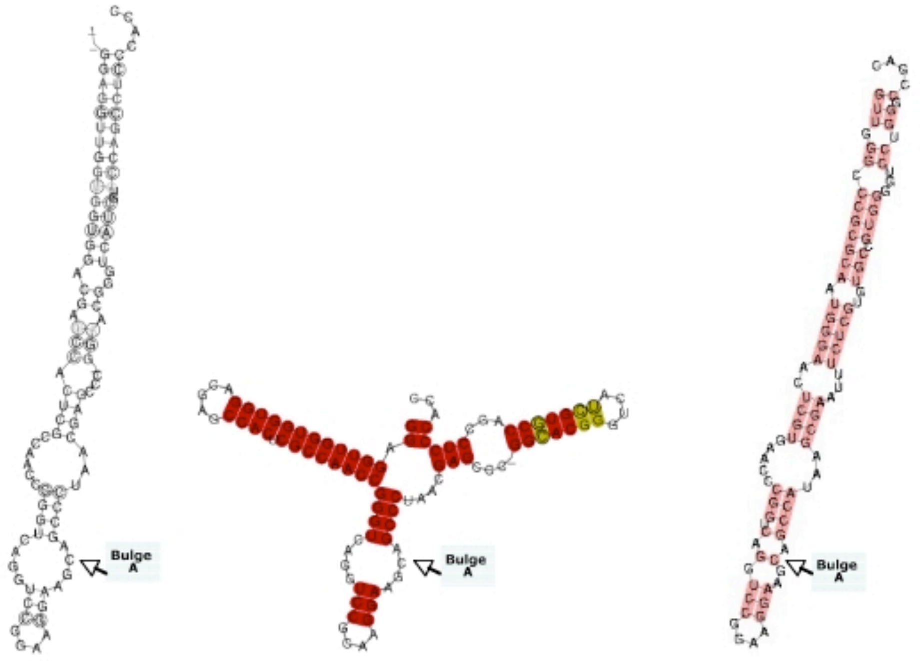


*Alifod prediction* *RNAz prediction* *RFAM*

*sra56 = 6S*

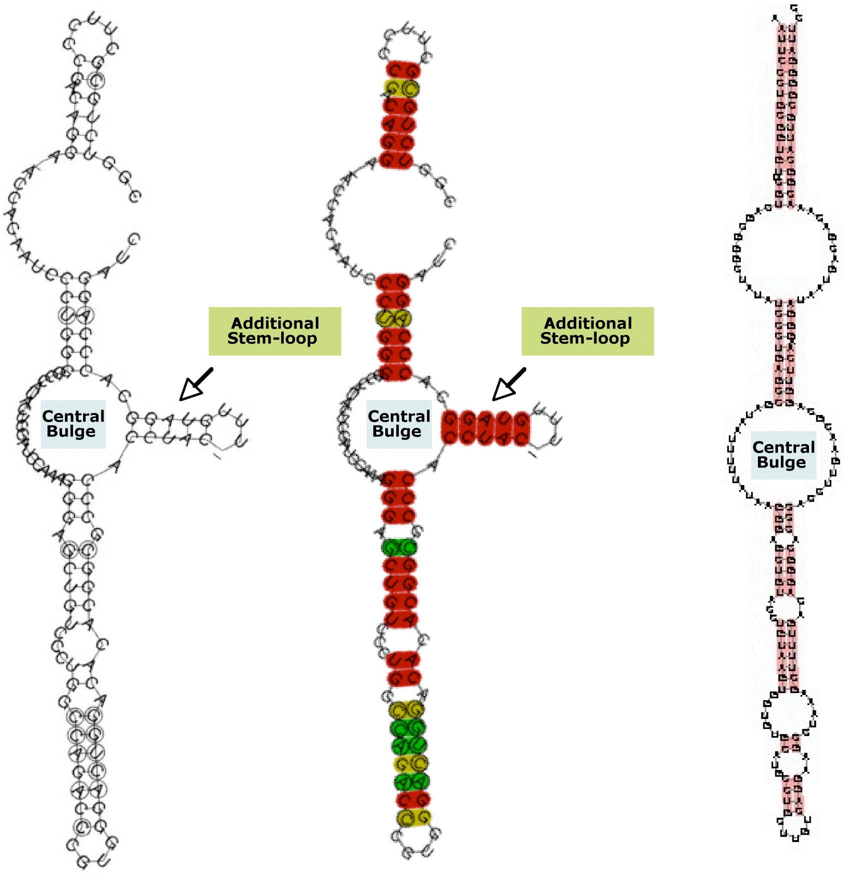


*Alifod prediction* *RNAz prediction* *RFAM*

*sra03*

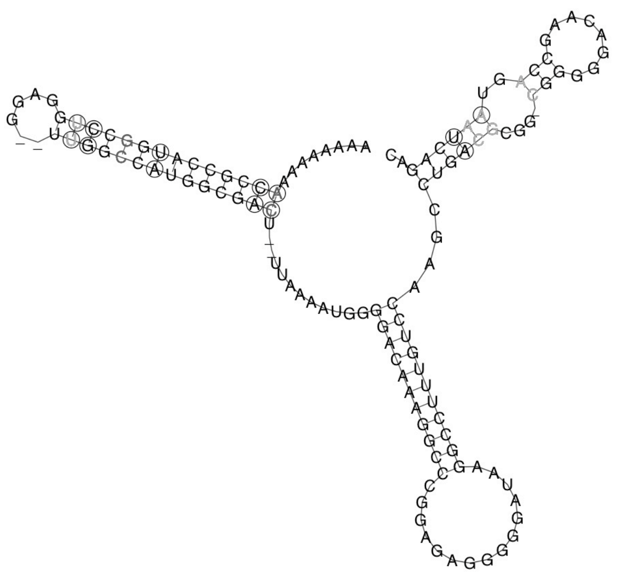

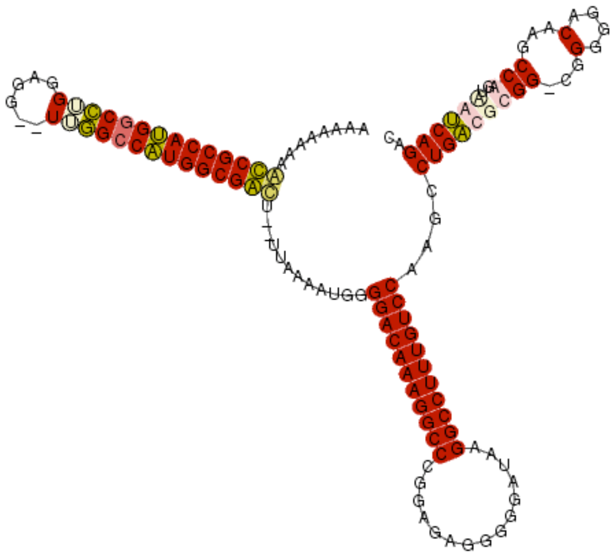

*Alifod prediction*

*RNAz prediction*

*sra25*

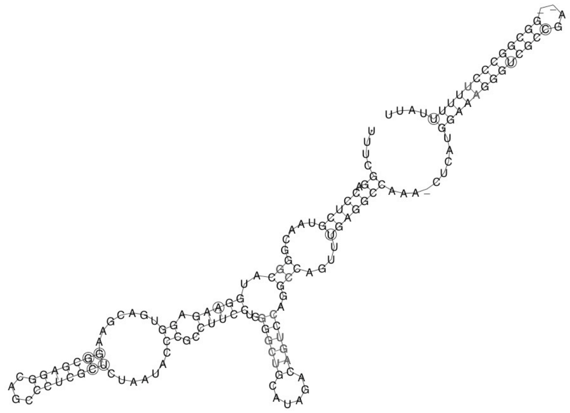

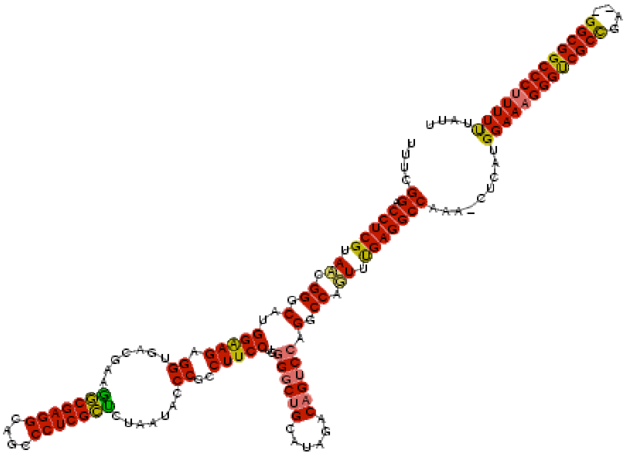

*Alifod prediction*

*RNAz prediction*

*sra33*

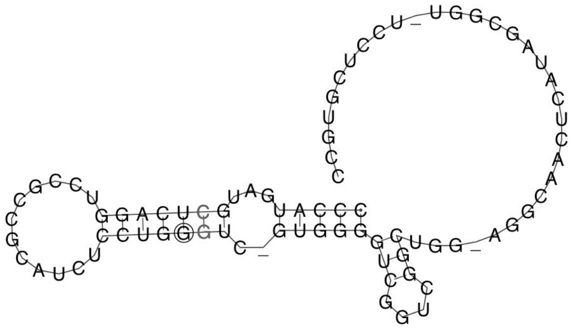

*Alifod prediction*

***No RNAz prediction (P > 0.5)***

*sra41*

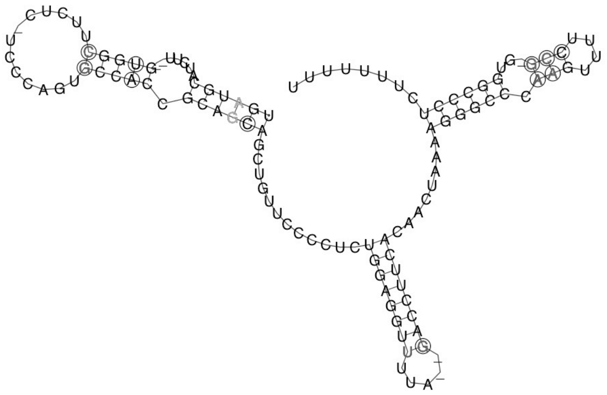

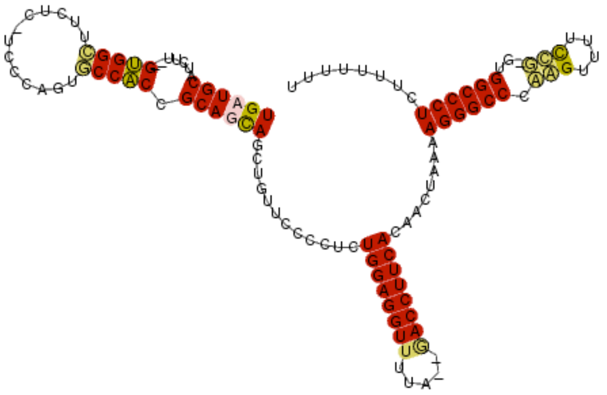

*Alifod prediction*

*RNAz prediction*

*sra32*

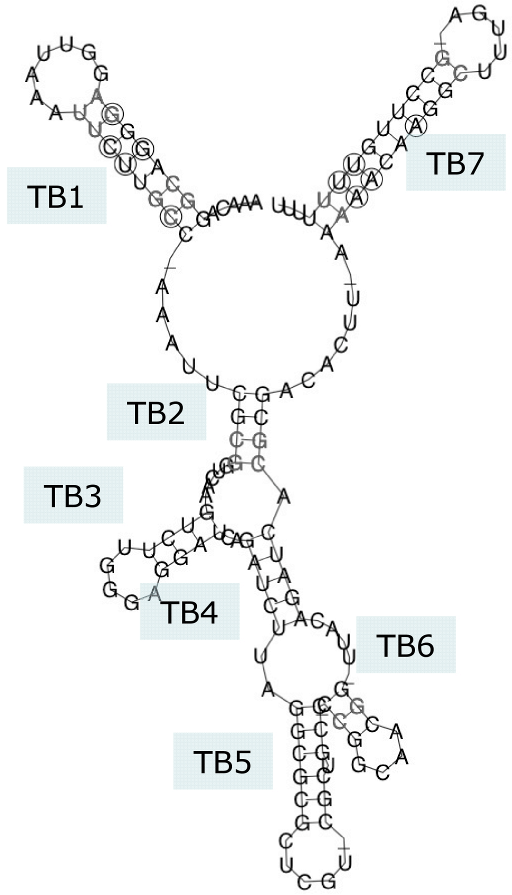


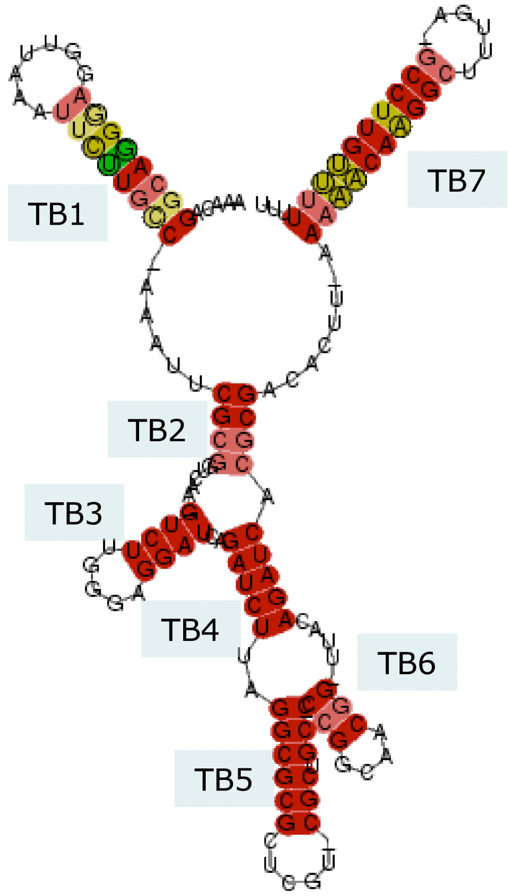


*Alifod prediction*

*RNAz prediction*
