# Supplementary material for: Identification of chromosomal alpha-proteobacterial small RNAs by comparative genome analysis and detection in Sinorhizobium meliloti strain 1021
Source: BMC Genomics. 2007 Dec 19;8:467. doi: 10.1186/1471-2164-8-467 (PMC2245857; doi:10.1186/1471-2164-8-467)
Supplement: Additional file 6 — sra41 multiple loci in S. meliloti and related alpha-proteobacteria. The data provided features the multiple loci of sra41 in subgroup-2 alpha-proteobacteria. [file 1471-2164-8-467-S6.pdf]

Loci	Organism	Replicon	Size (nt)	Coding
1	*S. meliloti*	chromosome	118	MHLWWLLLPVPPQQLFPSGGFNYLHNYKGPSFSCGPLF
2	*S. meliloti*	chromosome	112	-
3	*S. meliloti*	pSymA	109	MRMAVFLPVPLHQLFLSGGSYLQTHGSRFSDGPFL
1	*A. tum.*	chromosome	113	MHTVAFPPSSTASAVPLWRFLTFTLKGPWIYPAALFF
2	*A. tum.*	chromosome	114	-
1	*R. etli*	chromosome	113	MHMVAFPPSSTALAVPLWRFLTFTLIRALVFQWPSFF
2	*R. etli*	chromosome	112	MHMVAFPPSSTALAVPLWRFLTFTLIRALVFQWPSFF
3	*R. etli*	p42d	112	-
3	*R. etli*	p42a	111	MHMAAFLPVPPQQLFPSGGLLTFTLHGPRFPLAHFF
1	*R. legum.*	chromosome	113	MHMAAFLPVPPQQLFPSGGLLTFTLHGPRFPLAHFF
2	*R. legum.*	chromosome	113	-
3	*R. legum.*	pRL10	111	MMHMAVSSQCRRNSCSPLEVFDLHTSWASVLPTAHFF
3	*R. legum.*	pRL11	111	MQHGGFPPSSVVSAVPLWRFIPSQLMGHGSPSALFF
1	*M. loti*	chromosome	104	MHMAAFLPVPPQQLFPSGGLLTFTLHGPRFPLAHFF
2	*M. loti*	pMLA	107	-
3	*M. loti*	pMLA	66	-
1	*B. suis*	chromosome II	98	-
2	*B. suis*	chromosome I	119	-
