# Supplementary material for: Identification of chromosomal alpha-proteobacterial small RNAs by comparative genome analysis and detection in Sinorhizobium meliloti strain 1021
Source: BMC Genomics. 2007 Dec 19;8:467. doi: 10.1186/1471-2164-8-467 (PMC2245857; doi:10.1186/1471-2164-8-467)
Supplement: Additional file 7 — Strains used in this study.The data provided references the different bacteria used in this study. [file 1471-2164-8-467-S7.pdf]

Name	Provider	Reference Number	ATCC Number
S. meliloti	INRA Toulouse, France	Strain 1021	
S. meliloti	University of Rennes	Strain 102F34	
S. fredii	BCCM™, Belgium	LMG 6217	ATCC 35423
S. medicae	BCCM™, Belgium	LMG 18864	ATCC 700748
S. saheli	BCCM™, Belgium	LMG 7837	ATCC 51690
S. terangae	BCCM™, Belgium	LMG 7834	ATCC 700749
R. etli	BCCM™, Belgium	LMG 17827	ATCC 51251
R. leguminosarum biovar *phaseoli*	BCCM™, Belgium	LMG 4285	ATCC 14482
R. leguminosarum biovar *trifolii*	BCCM™, Belgium	LMG 4255	ATCC 10140
M. loti	BCCM™, Belgium	LMG 6125	ATCC 33669
B. japonicum	BCCM™, Belgium	LMG 6138	ATCC 10324
A. tumefaciens	Institut Pasteur, France	CIP 104333	ATCC 33970
