# Supplementary material for: Identification of chromosomal alpha-proteobacterial small RNAs by comparative genome analysis and detection in Sinorhizobium meliloti strain 1021
Source: BMC Genomics. 2007 Dec 19;8:467. doi: 10.1186/1471-2164-8-467 (PMC2245857; doi:10.1186/1471-2164-8-467)
Supplement: Additional file 8 — Oligonucleotides used in RACE and Northern assays. The data provided presents the oligonucleotide sequences used in RACE and Northern assays. [file 1471-2164-8-467-S8.pdf]

Name	Sequence 5’ → 3’	Specific use
sra32-5’	AAACAGGCAGGAAGACCAAA	RACE
sra32-3’	GGCCAAAGCCTTGTTTTTAAG	RACE
sra32NBrhiz	CGCGCCTAAGATCTTATCCTCCCAAG	Northern Blot
5S	GACCTACTCTCCCGCGTCTT	Northern Blot
rnpB	GCTTGCGGAGTAAGGGTGAA	Northern Blot
4.5S-ffs	AGGCTGGCACGATGACC	Northern Blot

Northern assays (dots and blots)

	Strand +	Strand -
sra01	TCACTTGCCGCTTCCTAATC	GATTAGGAAGCGGCAAGTGA
sra02	GTCCGACAAGACATCCCATT	AATGGGATGTCTTGTCGGAC
sra03	GTGAGTCTGGTGATTGGCCT	AGGCCAATCACCAGACTCAC
sra04	TCATCGGTATATATCGCGCC	GGCGCGATATATACCGATGA
sra05	AAGGAAGCAGCCCTAACGAG	CTCGTTAGGGCTGCTTCCTT
sra06	?	
sra07	AAAGTCAGCCTTTGCGTCAT	ATGACGCAAAGGCTGACTTT
sra08	GGGGCTTAACTTGCCTTACC	GGTAAGGCAAGTTAAGCCCC
sra09	TTCTTAACGCTGGAAATGGG	CCCATTTCCAGCGTTAAGAA
sra10	GAGCAGGAAGAAGGAGGCTC	GAGCCTCCTTCTTCCTGCTC
sra11	CAGTAGCGAAGGGCATCTTC	GAAGATGCCCTTCGCTACTG
sra12a	TAGTGTGGCGCGTTACTTTG	CAAAGTAACGCGCCACACTA
sra12b	GCTGTTCGATTGTACGGGTT	AACCCGTACAATCGAACAGC
sra13	TTGTTTAACGTGGCTGCAAG	CTTGCAGCCACGTTAAACAA
sra14	CGGAGCAGGTGATAAAGCTC	GAGCTTTATCACCTGCTCCG
sra15	AATTGAAAATCCGCGTATCG	CGATACGCGGATTTTCAATT
sra16	TCGACTTCTCCTCAAGGCAT	ATGCCTTGAGGAGAAGTCGA
sra17	GCAACTGATCTGCAACGAAA	TTTCGTTGCAGATCAGTTGC
sra18	CACTCTCTCGGCCAAAGAAC	GTTCTTTGGCCGAGAGAGTG
sra19	CCTTCTGCTTCTGGGTTGAG	CTCAACCCAGAAGCAGAAGG
sra20	CGCACTGAGGTCATTGTTTG	CAAACAATGACCTCAGTGCG
sra21	TGCTGCGTAACTTATACGCG	CGCGTATAAGTTACGCAGCA
sra22	AGAATTGGGAAGCCGGTC	GACCGGCTTCCCAATTCT
sra23	GGAGGTCAAGCTCTGGAATG	CATTCCAGAGCTTGACCTCC
sra24	GGGCAGAGGAAGGACTCTCT	AGAGAGTCCTTCCTCTGCCC
sra25	GACATGGGAGAGGTGAAGGA	TCCTTCACCTCTCCCATGTC
sra26	AATGAATCACAGGGCGATTC	GAATCGCCCTGTGATTCATT
sra27	GCGTTTAGTTGAAATCCGGA	TCCGGATTTCAACTAAACGC
sra28a	GCAACGAACCCAAAACATTT	AAATGTTTTGGGTTCGTTGC
sra28b	ATCCACGATTGTCATCGACA	TGTCGATGACAATCGTGGAT
sra29	AATGCCTTTACATTCCTCGC	GCGAGGAATGTAAAGGCATT
sra30	GCAATTTGTGACCACTCGTG	CACGAGTGGTCACAAATTGC
sra31	CGACATGCATGATAACGACC	GGTCGTTATCATGCATGTCG
sra32	CGCGTGATCTTTAATCCGTT	AACGGATTAAAGATCACGCG
sra33	CCCATGATGCTCAGGTCC	GGACCTGAGCATCATGGG
sra34	TACCTTCCGGTTCTGACGAC	GTCGTCAGAACCGGAAGGTA
sra35	GGGAGGTTTCCGACCTTAAG	CTTAAGGTCGGAAACCTCCC
sra36	TGGGTTCTTCAAGAGGATGG	CCATCCTCTTGAAGAACCCA
sra37	TAGGGACGAAGGCTAGTCGA	TCGACTAGCCTTCGTCCCTA

sra38	AGGAGTGTTGCCAATCCATC	GATGGATTGGCAACACTCCT
sra39	TCCTTTGGCCTGAGAGTTTC	GAAACTCTCAGGCCAAAGGA
sra40	GTCGCTCAGGTCTATAGCGG	CCGCTATAGACCTGAGCGAC
sra41	AGAAAAACTTGGGCCCTTGT	ACAAGGGCCCAAGTTTTTCT
sra42	GAGACAATGGCAGAGGCCTA	TAGGCCTCTGCCATTGTCTC
sra43	GGCGATTGTTGCAGAGAGAT	ATCTCTCTGCAACAATCGCC
sra44	GCACCGCTTATGTAATGGCT	AGCCATTACATAAGCGGTGC
sra45	TACTGGCTGACATCGTCGAG	CTCGACGATGTCAGCCAGTA
sra46a	CTGACTTCGGATCATCCGTT	AACGGATGATCCGAAGTCAG
sra46b	AGAACGTCCCCTTCACAGC	GCTGTGAAGGGGACGTTCT
sra47	CGTAAGCAGTACCGTGTCGA	TCGACACGGTACTGCTTACG
sra48	GATGACTGGCTGAGGGGTT	AACCCCTCAGCCAGTCATC
sra49	CTGAAGCACGTCTCGCTGC	GCAGCGAGACGTGCTTCAG
sra50	CCCACCCTTTCACCCTTACT	AGTAAGGGTGAAAGGGTGGG
sra51	TGAATACGCGAGTCAAAACG	CGTTTTGACTCGCGTATTCA
sra52	GGAATTCGCATCTTTAGATGG	CCATCTAAAGATGCGAATTCC
sra53	AGGTTCTTTCCTCCAGGGAA	TTCCCTGGAGGAAAGAACCT
sra54	TTCCCTCATGTATCGCATGA	TCATGCGATACATGAGGGAA
sra55	TGCCATATGTCGATAAGCCA	TGGCTTATCGACATATGGCA
sra56	AGGGACAGTCCCCTTGAGAT	ATCTCAAGGGGACTGTCCCT
sra57	ATTGGCTGAAGGTGTTGAGG	CCTCAACACCTTCAGCCAAT
sra58	GTGTATTCTCCCCCTTGTGG	CCACAAGGGGGAGAATACAC
sra59	TGTGCCTCTTCAGTAACCCC	GGGGTTACTGAAGAGGCACA
sra60	ATTATCGCGAAGGTAATGCG	ATTATCGCGAAGGTAATGCG
sra61	TTGTACTGTGCAGGTGAGGC	GCCTCACCTGCACAGTACAA
sra62	CAACTTCCGGTGCTCTTCTC	GAGAAGAGCACCGGAAGTTG
sra63a	GCAAGTAACGCCTGAGGAAC	GTTCCTCAGGCGTTACTTGC
sra63b	GAGGGGTCAATTCCTCGATT	AATCGAGGAATTGACCCCTC
sra63c	TTCTCAATCGCAATGGACTG	CAGTCCATTGCGATTGAGAA
sra63d	GAGAGCTGTAACCGCCACTC	GAGTGGCGGTTACAGCTCTC
sra64	GAAGCTTGTGCATTTTGCAA	TTGCAAAATGCACAAGCTTC
sra65a	ATCCGGATCTTCGCTAGGTT	AACCTAGCGAAGATCCGGAT
sra65b	ATCACGAGGGTGAACTGACC	GGTCAGTTCACCCTCGTGAT
sra65c	TTTTGCAGGTCTGCAACAAG	CTTGTTGCAGACCTGCAAAA
sra65d	TCCAGGATATAATCGGCCAG	CTGGCCGATTATATCCTGGA
sra65d	CTCTTCGAAGGCAAAGGAGA	TCTCCTTTGCCTTCGAAGAG
sra66	TGAAGATTGCACACTCGGAG	CTCCGAGTGTGCAATCTTCA
sra67	GCAAGGTTCAAAAGCCAGAC	GTCTGGCTTTTGAACCTTGC
