# Supplementary material for: Identification of chromosomal alpha-proteobacterial small RNAs by comparative genome analysis and detection in Sinorhizobium meliloti strain 1021
Source: BMC Genomics. 2007 Dec 19;8:467. doi: 10.1186/1471-2164-8-467 (PMC2245857; doi:10.1186/1471-2164-8-467)
Supplement: Additional file 9 — Oligonucleotides used in microarray design. The data provided presents the oligonucleotide sequences used in the microarray design. [file 1471-2164-8-467-S9.pdf]

Name	Sense	Sequence (5' -> 3')	IGR size	PCR size
5S	forward	CGACCTGGTGGTTCTGG	120	120
	reverse	AGACCTGGCAGCGACCTACT		
5Sint	forward	CGTTCCCATTCCGAACAC	120	76
	reverse	GACCTACTCTCCCGCGTCTT		
sra01	forward	TATCCAGTCCGCGTCAATCT	400	293
	reverse	TGATAAGGCGGGACAAAAAC		
sra02	forward	CAAAGGCTCCTGTCAGTTCC	306	161
	reverse	ACTTTCAGCCGAATCGTCAA		
sra03	forward	GGTCGGCCTATCCTTGAAAT	332	160
	reverse	CTCTTTGAAAGCGGGACAAA		
sra04	forward	GTCATCCGCAGCACCTTT	320	175
	reverse	GCCACCTGTCCATCCTTCT		
sra05 (*4.5S*)	forward	AGGCTTGGCTTGCATTTG	161	101
	reverse	CTGCTTCCTTCCGGACCT		
sra05Bis (*4.5S*)	forward	GCATTTGCTGCGGATATTG	161	148
	reverse	CGACAGGACGGAGAAGGTG		
sra06	forward	GTATTGTAAAGGGCGCCACA	227	199
	reverse	GGGAGGCTTCAAAGAAAACC		
sra07	forward	GGAGGGGCTACTTCTGCC	162	138
	reverse	GATGAGGCCCCTCCCTAA		
sra08	forward	CTTGCCTTACCGTCACCG	214	144
	reverse	CACCGCGTCTTCTTCGAC		
sra09	forward	GGGCGGCTTTCCTTTTT	180	133
	reverse	TCCAGCGTTAAGAACGGC		
sra10A	forward	GATTGACCTTGCCACGCT	448	388
	reverse	AAAGCCCAAAGTCACCCC		
sra10B	forward	CGAGACGATAAAACCGGG	417	365
	reverse	TGGCGGCCTCATCTTTTA		
sra11	forward	ATTACTGCCCAGCCAACATC	179	160
	reverse	AAGAAATGCCTTCGCTTTCA		
sra12A	forward	GCTGGCACTAGCAGTCCTTC	689	178
	reverse	TCGTCGCGAGACACTTTATG		
sra12BFAR	forward	TAGTGTGGCGCGTTACTTTG	689	550
	reverse	TCGTCGCGAGACACTTTATG		
sra13	forward	CGAAACGACCGGTTTGAC	137	130
	reverse	CCAAACTCCGGGAATTGA		
sra14A	forward	CGGAGCAGGTGATAAAGCTC	773	230
	reverse	GGGTGTACTTGGCGTCAGTT		
sra14B	forward	CAAAGACACGCATTCCATCA	773	567
	reverse	CATCCATTGCAAAACGGTTA		
sra15	forward	AGCTTAGCGGGTGCATCG	210	150
	reverse	CACGCGAATGAGGCTGT		
sra16	forward	GAATCGCAGACGACACAAGA	566	216
	reverse	CCGGAATCGCTAAAATTCAA		
sra17A	forward	GTTACTCCGCGTTGTTTGCT	686	211
	reverse	TTTCGTTGCAGATCAGTTGC		
sra17BFAR	forward	TTTCGTTGCAGATCAGTTGC	686	385
	reverse	GGCCTTCCGGGAGTATTG		
sra17AFBR	forward	CGAGTATCAGGACGCGAATC	686	456

	reverse	GTTACTCCGCGTTGTTTGCT		
sra18	forward	CTCTCGGCCAAAGAACCA	247	190
	reverse	CGGTAATCTTGCGTGGGA		
sra19	forward	CCTTCTGCTTCTGGGTTGAG	449	213
	reverse	TCGGGGTCTTTCACTTGTCT		
sra20	forward	CATTAACATTTGCCGGGTG	139	99
	reverse	TGCAGCGGATCTGAGACA		
sra21	forward	AATCACGGCATTGTGATCG	234	154
	reverse	GAAAGCATTCCGGACTTCAA		
sra22A	forward	TCGATCAGACCCTTGTGTTG	497	247
	reverse	GGCACCTTGAGTGTCCTCAT		
sra22B	forward	TTTCTGGAATCGGATGTCGT	497	249
	reverse	TTTCTGCTTCAAGGCTGGTT		
sra23	forward	CCAGTGAGGTTTCGCCAT	179	101
	reverse	AGAGCTTGACCTCCGCCT		
sra24	forward	TCCCTGCCGATCTAAGAATG	446	214
	reverse	AATGCCATTCACTCCTCAGC		
sra25	forward	CGTCAACGAACAGAAAGGAAG	277	154
	reverse	ATGAGCTTTGGCCTCACAAT		
sra26	forward	AACGTCGATCCCATCTTCAG	222	212
	reverse	TCAACGCATCAATCCATCTC		
sra27	forward	TTGAAATCCGGACGTGAAAT	444	181
	reverse	CGCTCGTCTTATGGATGTGA		
sra28A	forward	CGAACCCAAAACATTTCGTC	907	254
	reverse	TAATAGGTGGGCTCGCTGGT		
sra28B	forward	CGATCGTTCCTCCAACAACT	907	298
	reverse	GCCGCCATTATTGTGTTTTT		
sra29	forward	TGAGAGCCGGCAAGAAAG	283	198
	reverse	GACACCAATCCGACGAGG		
sra30	forward	GACGCGCGGGAACAGTAG	164	150
	reverse	ATGATGCGCCGGTTTTGT		
sra31	forward	ATGCCGCCCAAAAGTGT	96	89
	reverse	CTGAGGCCTAGGACATAAAGC		
sra32A	forward	GAACTGCTTCGCCGTAATGT	570	195
	reverse	ATTTCGGCTCACAAAACAGG		
sra32B	forward	TTGGTCTTCCTGCCTGTTTT	570	249
	reverse	AGCGTTCATAGCCGCTTC		
sra33	forward	GCCGGGGTAAGCACATC	346	245
	reverse	GGTTGCAGCACGAGGAAT		
sra34	forward	ACGCTTTTTGAAGCGGTG	287	197
	reverse	GTGAAACCTTTGCGCGTT		
sra35	forward	GCGTAAGCCAATAGGAATGG	221	207
	reverse	TCTGCCTTTCACTGCTCCTT		
sra36	forward	CGCCTCTCTTTGACCGTT	205	172
	reverse	CGACCCCGTCAATCAAT		
sra37	forward	CGATTCAGCAACACATTTCC	230	154
	reverse	TGACGGCACACAAACTCACT		
sra38A	forward	GTTTGAGCCGGGATTACAGA	1338	217
	reverse	GCCTCAACGCCATCATCTAT		
sra38D	forward	AATAATGGGCAAGTGGATCG	1338	449

	reverse	GCTTGAGGCTCCTTAGCAGA		
sra39A	forward	GACAAATTCGAGCCCCCT	448	409
	reverse	TATGGGCGAAGGACAGGA		
sra39B	forward	TATCTTCTCCCCGACGGC	366	310
	reverse	CATAGCGGCGCAAGAAAT		
sra40A	forward	ATACGGGAGCTGCACTTGAT	908	161
	reverse	CGATTGCCCGCATAGAGTAT		
sra40B	forward	TTTGTGTACCGTCGGTTGAA	908	371
	reverse	GACAGCCCTCTTGCGATG		
sra41	forward	AGGAAGCGGGCCATAAAC	592	396
	reverse	ATGATTGCCGATAGCCCA		
sra42	forward	AATGGCAGAGGCCTACCC	327	277
	reverse	TCTCCCAAGAGCGAGGTG		
sra43AFBR	forward	TACATCTTTCCCCGCATTTC	765	550
	reverse	CGTTACCTGAGTGAAATATACC		
sra43BFAR	forward	GGAGTTGAGCGATGAACCAG	765	445
	reverse	CCAGCCAAGAGCCTTATGAC		
sra44	forward	CGGCAGCTTGTCAATACG	94	81
	reverse	CTTGGCAAATGGGGTGAA		
sra45	forward	GTAAACCGGCAAAGAAGCAG	274	164
	reverse	ACCACCCAATCGCAAAATTA		
sra46	forward	CGCCTTCCCGAATAGCTT	237	157
	reverse	ATATGCCGCCGTCAGGT		
sra47AFBR	forward	GAAGAATGCTCCGGAATCAA	647	450
	reverse	CCTGGGTCACAGTAGCGAAT		
sra47BFAR	forward	CTTCGCCTGCCTCTTCGT	647	307
	reverse	GCGCTAGAGAGTCCTTCGTG		
sra48	forward	GAATCCACAGGGGCGAC	411	339
	reverse	CTCTGGCTGGAACGCCT		
sra49 (tmRNA)	forward	[39]		206
	reverse	[39]		206
sra50A	forward	CCGCACATACACTCGCAC	377	350
	reverse	GGCAAAGCACGTTTCCTG		
sra50B	forward	TCTGTATGGCTCCGCCTC	377	396
	reverse	CGCCGGTCGTTTAATGAG		
sra51A	forward	TGAATACGCGAGTCAAAACG	529	250
	reverse	ATTGAGGAAAGGGTCCTGGT		
sra51B	forward	TTGAGCCGGCAATATATCCT	529	281
	reverse	TTGCGTCTTCTCATTGCTCA		
sra52	forward	TGATCTCCAACAACCCGAAT	273	208
	reverse	TATCGCGTGACAAAACCAAA		
sra53A	forward	AGAAATGAACCACGCCTCAC	881	334
	reverse	ATGGCTGACAGAGGACCAAC		
sra53B	forward	CCTCTGTCAGCCATTGGAGT	881	324
	reverse	TTTCCTTATTCGCCGTTTTG		
sra54	forward	GCATCCCGCTCTAACTCCTT	410	238
	reverse	ACTCGTAAATGCGCGAAAGT		
sra55	forward	CGCACGGTCGTGAGTAAGAT	224	159
	reverse	GAGTGCACCTTATCGGCTTC		
sra56	forward	GTCCGGCCAGGGACAGT	224	153

	reverse	TGCGATTTCGCAGAAATTAT		
sra57	forward	CAAGCAACTGAAGCCGAGAC	292	170
	reverse	CCTCAACACCTTCAGCCAAT		
sra58	forward	CTCGCTCTGACCAAGCATTC	234	151
	reverse	GGTGCTTCCCTTCTTGACAC		
sra59	forward	TTCCGGCAATGAAGGTGT	526	364
	reverse	GGTAGACGGGTCTCCGGT		
sra60A	forward	CCCTTGGTTTTTACGGGTTT	944	341
	reverse	AACGGAGTGGCGAATACTTG		
sra60B	forward	CACGACTCCGATCAACATTC	944	277
	reverse	TCCGATCTTCTACGCGAAAC		
sra61	forward	TTTGGCTTATCCCATTGAGC	359	207
	reverse	CATATTGCTCCAGCCACTCA		
sra62A	forward	CGCGCTCGAACTAAGATTG	721	337
	reverse	ACGTGAAAGAGCCCAAAAAG		
sra62B	forward	ACGTAAGGCGTCAGCTTTGT	721	321
	reverse	AAGCCAAACCACAGTCCATC		
sra63A	forward	AATGCTCTTCACTGCCATCC	2018	350
	reverse	GCTTTCACTGCCATCTGCAC		
sra63B	forward	GCCAAATCGTTCGGTAACAT	2018	350
	reverse	GACGTCACCAGCCTGTTCTC		
sra63C	forward	CGATGCAAGGCTGAAGAAAC	2018	321
	reverse	ACCGGTATCCTGCGATATGA		
sra63D	forward	GAGATCGAAGGCACTCATCC	2018	329
	reverse	GACGTCCTCAAGGCCAGA		
sra64	forward	CTTGGTTTTGGACCAAGCAT	592	349
	reverse	GCGTGGAAGTCAGCCACTAT		
sra65A	forward	GCTTCACGTGTCACGATTTC	964	340
	reverse	GATGTAGTCCGCTTGCCTTG		
sra65B	forward	TCCGTAAACTGGCAGCAGTA	964	350
	reverse	CCAGAGGGCAGGTGAGAATA		
sra66	forward	TGCACGAAGCCAATGTAGAC	728	236
	reverse	CTCCGAGTGTGCAATCTTCA		
sra67	forward	GCACCGCAAGGTTCAAAA	70	54
	reverse	GTTGGGTATCGCCCCATC		
sra67Bis	forward	CCGGTAGCGCTCTAAAACAG	135	129
	reverse	GGTTCTTCCTCCAGACATGC		
